# Immunohistochemical Evaluation of Non-Thermal Plasma-Treated Xenogenic Bone Graft for Enhanced Bone Regeneration in a Rabbit Calvarial Defect Model

**DOI:** 10.3390/jfb17100503

**Published:** 2026-10-06

**Authors:** Hyung-Gyun Kim, Young-Suk Moon, Hyunsuk Choi, Dong-Seok Sohn

**Affiliations:** 1 Department of Dentistry and Advanced General Dentistry, Daegu Catholic University School of Medicine, Daegu 42472, Republic of Korea; hgkim25@cu.ac.kr; 2 Department of Anatomy, Daegu Catholic University School of Medicine, Daegu 42472, Republic of Korea; ysmoon@cu.ac.kr; 3 Department of Dentistry and Prosthodontics, Daegu Catholic University School of Medicine, Daegu 42472, Republic of Korea; hschoi@cu.ac.kr; 4 Department of Dentistry and Oral and Maxillofacial Surgery, Daegu Catholic University School of Medicine, Daegu 42472, Republic of Korea

**Keywords:** non-thermal plasma, xenogenic bone graft, bone regeneration, immunohistochemistry, osteogenesis, angiogenesis, rabbit calvarial defect

## Abstract

Bone graft materials may undergo biological aging during storage, potentially reducing surface hydrophilicity and early cellular responses. Non-thermal plasma treatment has been investigated as a surface modification strategy to improve the biological performance of graft materials. This study evaluated osteogenic and angiogenic responses associated with non-thermal plasma-treated xenogenic bone grafts using tissues obtained from an 8-mm rabbit calvarial defect model. Twenty-four adult male New Zealand white rabbits were assigned to 2-, 4-, and 8-week healing periods, and defects were filled with untreated or plasma-treated xenografts. Immunohistochemical expressions of PCNA, VEGF, PECAM-1, collagen I, collagen III, ALP, BMP2, BMP4, BMP7, osteocalcin, and osteopontin were assessed semiquantitatively. Most markers showed greater expression in the experimental group during the early and intermediate healing stages. VEGF and PECAM-1 showed significant intergroup differences at 2 and 4 weeks but not at 8 weeks, whereas several osteogenic markers showed lower expression in the experimental group at 8 weeks. These temporal expression patterns suggest that non-thermal plasma treatment is associated with earlier activation of biological responses related to cell proliferation, angiogenesis, and osteogenesis, followed by changes consistent with later tissue maturation and remodeling.

## 1. Introduction

Tooth loss is a common clinical condition caused by trauma, periodontal disease, and infection. Traditionally, missing teeth have been restored with conventional prosthetic treatments, such as crowns, fixed dental prostheses, and removable dentures. Although these approaches remain widely used, implant-supported rehabilitation has become a well-established treatment option for restoring edentulous sites since the introduction of dental implants, with long-term success rates exceeding 90% [1].

However, after tooth loss, the mechanical intraosseous stimulation normally transmitted to the alveolar bone through the periodontal ligament fibers is lost, leading to continuous remodeling and resorption of the alveolar ridge. Consequently, the residual alveolar bone may become insufficient in volume and dimensions to allow proper implant placement and achieve adequate primary stability [2,3].

To overcome this clinical limitation, alveolar ridge augmentation may be required at sites with insufficient bone volume to restore adequate ridge dimensions, either before implant placement or during the implant placement procedure. Bone regeneration relies on three fundamental mechanisms: osteogenesis, in which osteogenic cells within the graft directly form new bone; osteoinduction, in which signaling factors stimulate undifferentiated cells to differentiate into osteogenic cells; and osteoconduction, in which the graft serves as a scaffold that allows bone ingrowth from adjacent host tissues. An optimal bone graft material should support these regenerative processes while providing good biocompatibility and sufficient structural stability [4,5,6].

In clinical practice, four major categories of bone graft materials are used: autografts, allografts, xenografts, and alloplasts. Autografts have long been regarded as the gold standard for bone grafting because they provide osteogenic, osteoinductive, and osteoconductive properties and carry a low risk of immunologic rejection. However, their use requires a separate donor site, is limited by the amount of bone available for harvesting, and carries the risk of donor-site pain, morbidity, and additional surgical burden. As alternatives to autogenous bone, allografts, xenografts, and various synthetic bone substitutes have been developed and incorporated into clinical use [6,7]. Xenografts, however, exhibit less osteoinductive potential than autografts and some allografts, and undergo relatively slow remodeling and resorption in vivo. Nevertheless, xenografts are widely used in dental practice because of their ready availability, the absence of donor-site surgery, and their ability to serve as an osteoconductive scaffold while maintaining structural stability within the bone defect over an extended period [4,5].

Commercially available bone graft materials, including xenografts, are rarely used immediately after production and typically undergo sterilization, distribution, and a period of storage before clinical use. During this time, the materials are exposed to ambient air, allowing atmospheric hydrocarbons to gradually accumulate on their surfaces, a process known as biological aging. This hydrocarbon buildup can reduce surface hydrophilicity and interfere with protein adsorption and early cell attachment, potentially compromising the initial biological interactions between osteogenic cells and the graft material [8,9].

To address the adverse effects of biological aging, various surface modification techniques have been investigated to remove accumulated hydrocarbons and restore bioactivity without altering the physical structure of bone graft materials. Among these approaches, ultraviolet (UV) irradiation and plasma treatment have been widely studied. Non-thermal atmospheric-pressure plasma has attracted considerable attention for surface processing of dental implants and bone graft materials because it operates at lower temperatures. Plasma, an ionized gas generated by applying energy, produces reactive oxygen and nitrogen species (RONS). Upon exposure of biomaterial surfaces to these reactive species, hydrocarbon contaminants are effectively decomposed, and surface wettability is markedly enhanced. These surface alterations create a favorable microenvironment for protein adsorption and for cellular attachment, proliferation, and differentiation. Evidence from numerous studies demonstrates that plasma treatment enhances these early biological responses and subsequently promotes bone regeneration [8,9,10,11,12].

The beneficial effects of plasma treatment on bone regeneration were also demonstrated in our previous study. Histological and histomorphometric analyses of non-thermal plasma-treated xenografts in an 8-mm rabbit calvarial defect model showed significantly higher proportions of new bone and vascular areas with plasma treatment compared with the control group across all observation periods. Graft resorption and bone marrow formation were also enhanced [13]. These findings suggest that plasma treatment may favorably influence the regenerative process surrounding xenografts, including bone formation and angiogenesis. However, histological and histomorphometric analyses alone provide limited insight into the cellular and molecular mechanisms underlying these regenerative effects. Bone regeneration is a complex process involving coordinated osteogenic cell differentiation and maturation, extracellular matrix formation and mineralization, angiogenesis, and bone remodeling. Therefore, assessing osteogenic and angiogenic marker expression is necessary to better understand how plasma treatment enhances bone regeneration.

Accordingly, this study aimed to immunohistochemically evaluate osteogenic and angiogenic marker expression associated with non-thermal plasma-treated xenografts, thereby complementing our previous histological findings and providing further insight into the mechanisms underlying plasma-enhanced bone regeneration.

## 2. Materials and Methods

### 2.1. Experimental Materials

This study used the same xenogenic bone graft material (SANTA-OSS^®^, BIOTEM Co., Ltd., Hanam-si, Gyeonggi-do, Republic of Korea) and non-thermal atmospheric pressure plasma device (ACTILINK™ Reborn, Plasmapp Co., Ltd., Daejeon, Republic of Korea) as in our companion study [13]. As previously described, the graft material was treated with non-thermal plasma in two consecutive 1-min cycles immediately before grafting, in accordance with the manufacturer’s protocol [13].

### 2.2. Surgical Procedures

**Figure 1:**
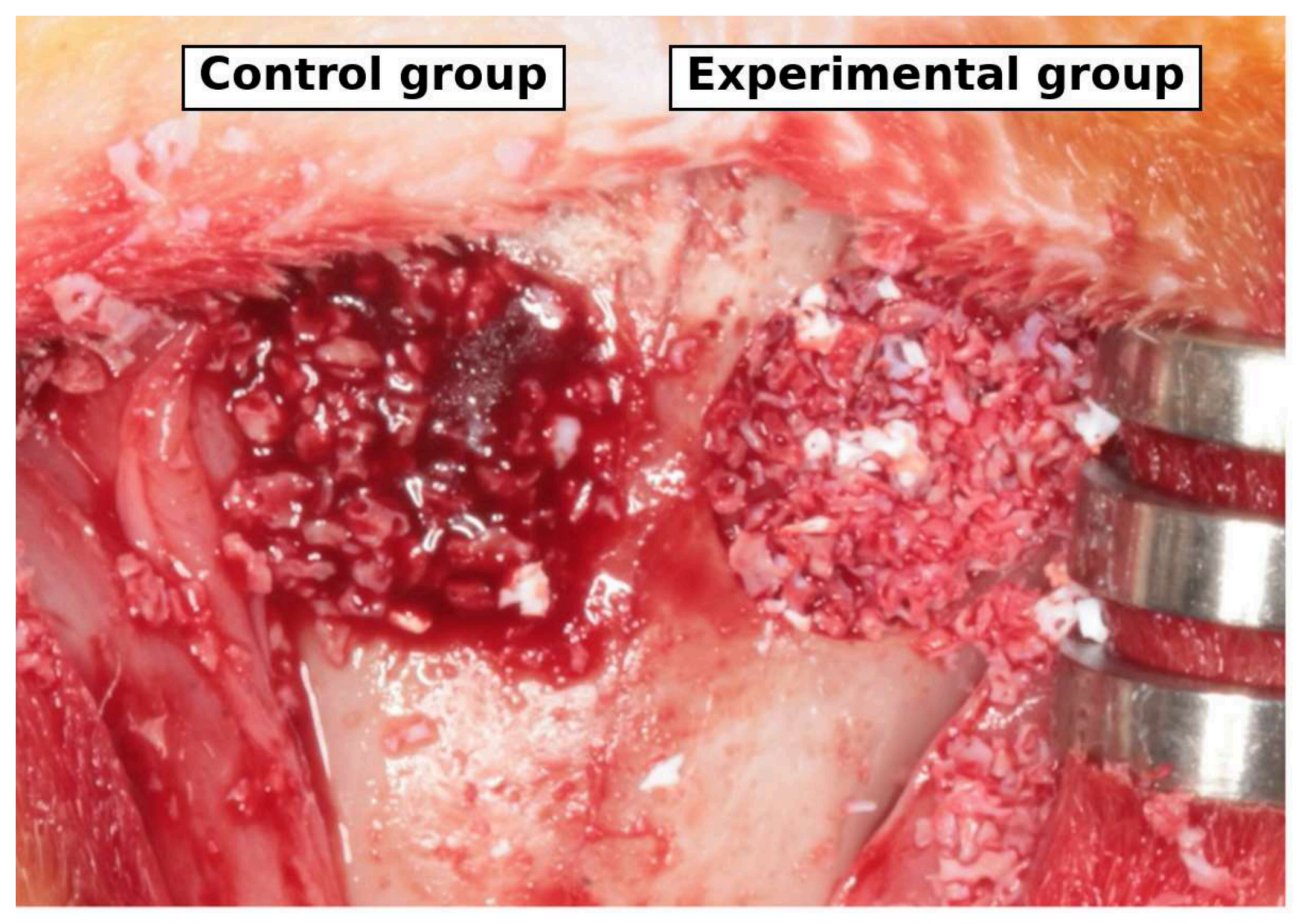
Bone graft placement in rabbit calvarial defects. The left defect received untreated SANTA-OSS (Control group), whereas the right defect received non-thermal plasma-treated SANTA-OSS (Experimental group). Adapted from Ref. [13].

This study used tissue specimens from the same animal experiment reported in our companion study [13]. Briefly, 24 adult male New Zealand white rabbits were divided into three groups according to healing duration, with eight animals allocated to each of the 2-, 4-, and 8-week periods. Throughout the experimental period, housing conditions remained consistent for all animals, including the same commercial diet, a temperature of 22 ± 2 °C, relative humidity of 50 ± 10%, and a 12-h light/dark cycle, as previously described [13]. Surgical procedures were performed under general anesthesia induced by intramuscular administration of ketamine (30 mg/kg, ketalar; Yuhan Co., Seoul, Republic of Korea) and xylazine (10 mg/kg, Rompun; Bayer Korea, Seoul, Republic of Korea), with local anesthesia using lidocaine containing epinephrine, as previously described [13]. Two bilateral 8-mm defects were created in each rabbit calvarium. The control site received 0.5 cc of untreated xenogenic bone graft, while an equal volume of plasma-treated graft material was placed in the contralateral experimental site (Figure 1). Control and experimental sites were assigned randomly. Animals were sacrificed at 2, 4, or 8 weeks after surgery, and calvarial specimens were harvested for subsequent analysis. All animal procedures followed a protocol that received approval from the Institutional Animal Care and Use Committee of Daegu Catholic University Medical Center (DCIAFCR-240620-12-Y), as previously reported.

### 2.3. Tissue Preparation

Tissue specimens were processed according to the protocol described in our companion study [13]. Briefly, the harvested specimens underwent fixation with 10% neutral buffered formalin at room temperature for 24 h, followed by decalcification with 10% formic acid for 10 days, and paraffin embedding (Paraplast^®^; Sigma-Aldrich Co., Oxford, MS, USA). Serial sections (5-µm-thick) were prepared along a plane passing through the center of each defect for immunohistochemical analysis.

### 2.4. Immunohistochemical Measurements

Immunohistochemical analysis was performed using antibodies against proliferating cell nuclear antigen (PCNA; mouse monoclonal, PC10, Santa Cruz Biotechnology, Dallas, TX, USA; 1:200), vascular endothelial growth factor (VEGF; rabbit polyclonal, A-20, Santa Cruz Biotechnology, Dallas, TX, USA; 1:100), platelet endothelial cell adhesion molecule-1 (PECAM-1; goat polyclonal, M-20, Santa Cruz Biotechnology, Dallas, TX, USA; 1:100), collagen I (rabbit monoclonal, ab138492, Abcam, Cambridge, UK; 1:100), collagen III (mouse monoclonal, FH-7A, Abcam, Cambridge, UK; 1:500), alkaline phosphatase (ALP; rabbit monoclonal, ab307726, Abcam, Cambridge, UK; 1:500), bone morphogenetic protein 2 (BMP2; mouse monoclonal, ab6285, Abcam, Cambridge, UK; 1:200), BMP4 (rabbit polyclonal, ab39973, Abcam, Cambridge, UK; 1:500), BMP7 (rabbit polyclonal, ab84684, Abcam, Cambridge, UK; 1:500), osteocalcin (rabbit polyclonal, ab198228, Abcam, Cambridge, UK; 1:200), and osteopontin (mouse monoclonal, ab69498, Abcam, Cambridge, UK; 1:100).

Paraffin-embedded sections were processed and washed with phosphate-buffered saline (PBS). For antigen retrieval, the sections were incubated in citrate buffer (pH 6.0) at 95 °C for 30 min. Endogenous peroxidase activity was blocked with 0.3% H_2_O_2_ in PBS for 30 min, followed by blocking of nonspecific protein binding with PBS containing 10% normal horse, goat, or rabbit serum (Vector Laboratories, Newark, CA, USA) for 30 min, as appropriate. Negative controls were included for each antibody. No problematic background or nonspecific staining that interfered with interpretation was observed, including when rabbit-derived primary antibodies were applied to rabbit tissue. The sections were then incubated with the respective primary antibodies for 2 h at room temperature and washed three times with PBS. Subsequently, the appropriate biotinylated secondary antibody (1:100; anti-mouse IgG, anti-rabbit IgG, or anti-goat IgG; Vector Laboratories, Newark, CA, USA) was applied for 1 h at room temperature, followed by three washes with PBS. An avidin–biotin–peroxidase complex (ABC; Vector Laboratories, Newark, CA, USA) was then applied for 1 h. After washing, immunoreactivity was visualized using 0.05 M Tris-HCl (pH 7.6) containing 0.01% H_2_O_2_ and 0.05% 3,3′-diaminobenzidine (DAB; Sigma-Aldrich Co., Oxford, MS, USA). The sections were counterstained with hematoxylin and mounted.

We examined the stained sections using an Axiophot photomicroscope (Carl Zeiss, Jena, Germany) with AxioVision SE64 software (version 4.9.1, Carl Zeiss, Jena, Germany). At each healing period, we randomly selected 20 microscopic fields at ×100 magnification from the eight specimen slides in each group for immunohistochemical evaluation. Because of the limited size of each defect/specimen, only approximately two to three microscopic fields could be selected from each specimen. The defect/specimen from each rabbit, rather than each individual microscopic field, was treated as the experimental unit for the statistical analysis. Immunostaining was evaluated semiquantitatively using a five-point scale ranging from 0 to 4. Three histopathology experts independently performed semiquantitative immunohistochemical scoring and were blinded to the experimental groups. The evaluation was performed twice, and the three examiners’ scores were combined and averaged to obtain the final values. For PCNA, VEGF, PECAM-1, and ALP, scores were assigned according to the proportion of positively stained cells: 0, no positive cells; 1, 1–10%; 2, 11–33%; 3, 34–66%; and 4, 67–100%. For collagen I, collagen III, BMP2, BMP4, BMP7, osteocalcin, and osteopontin, staining intensity was scored as follows: 0, negative; 1, weak but detectable; 2, mild; 3, moderate; and 4, strong.

### 2.5. Statistical Analysis

Because this study used tissue specimens obtained from the same animal experiment as our companion study [13], no additional sample size calculation was performed. The sample size for the original animal experiment was determined using G*Power software (version 3.1), as previously described [13]. Based on previous rabbit calvarial defect studies, an effect size of 1.5 was assumed for the percentage of newly formed bone area, with α = 0.05 and statistical power (1 − β) = 0.80. The calculation indicated a minimum of six animals; eight animals were therefore included at each healing period to allow for potential dropout. Data are expressed as mean ± standard error. Statistical analyses were performed using SPSS software (version 25.0, IBM Corp., Chicago, IL, USA). Differences between the control and experimental groups at each healing period, as well as differences among healing periods within each group, were evaluated using one-way analysis of variance (ANOVA) followed by Tukey’s post hoc test. Although the control and experimental defects were created contralaterally within the same animal, the within-animal pairing was not separately modeled in the statistical analysis. A *p*-value < 0.05 was considered statistically significant.

## 3. Results

### 3.1. Expression of PCNA

**Figure 2:**
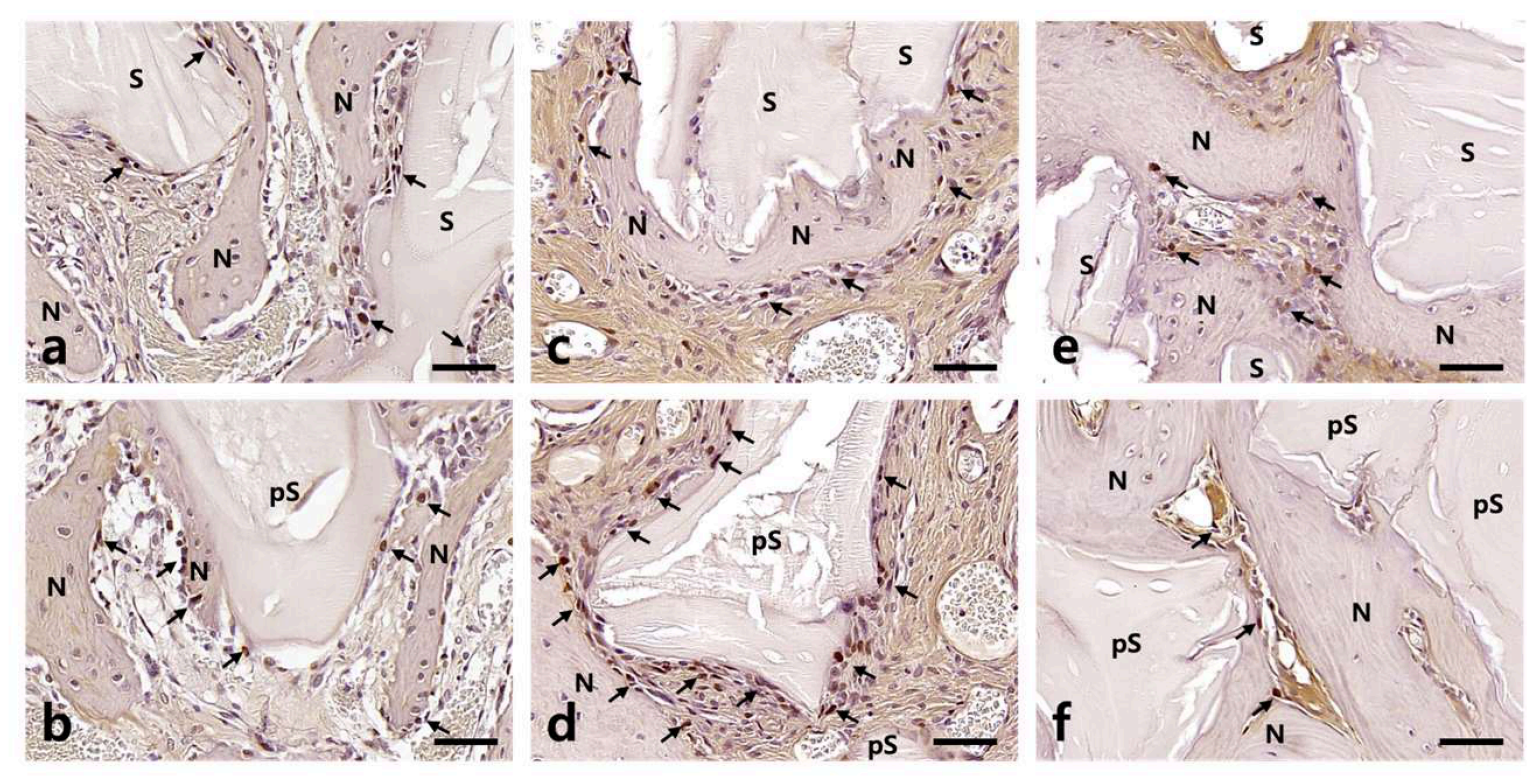
Immunohistochemical staining for PCNA in control (**a**,**c**,**e**) and experimental (**b**,**d**,**f**) groups at 2 (**a**,**b**), 4 (**c**,**d**), and 8 (**e**,**f**) weeks after surgery. PCNA-positive cells are indicated by arrows. Abbreviations: N, newly formed bone; S, SANTA-OSS particles; pS, plasma-treated SANTA-OSS particles (Scale bar: 50 μm).

PCNA immunoreactivity varied according to healing period in both groups. In the control specimens, PCNA-positive cells were mainly detected along the surfaces of the SANTA-OSS particles and within the connective tissue at 2 and 4 weeks, with more prominent staining at 4 weeks (Figure 2a,c). By 8 weeks, staining had decreased, and positive cells were observed along newly formed bone (Figure 2e). In the experimental specimens, PCNA-positive cells were identified around the plasma-treated SANTA-OSS particles and newly formed bone at 2 weeks (Figure 2b). At 4 weeks, positive cells were present around the graft particles and newly formed bone and within the connective tissue (Figure 2d), whereas at 8 weeks they were mainly associated with the graft particles and newly formed bone (Figure 2f).

**Figure 3:**
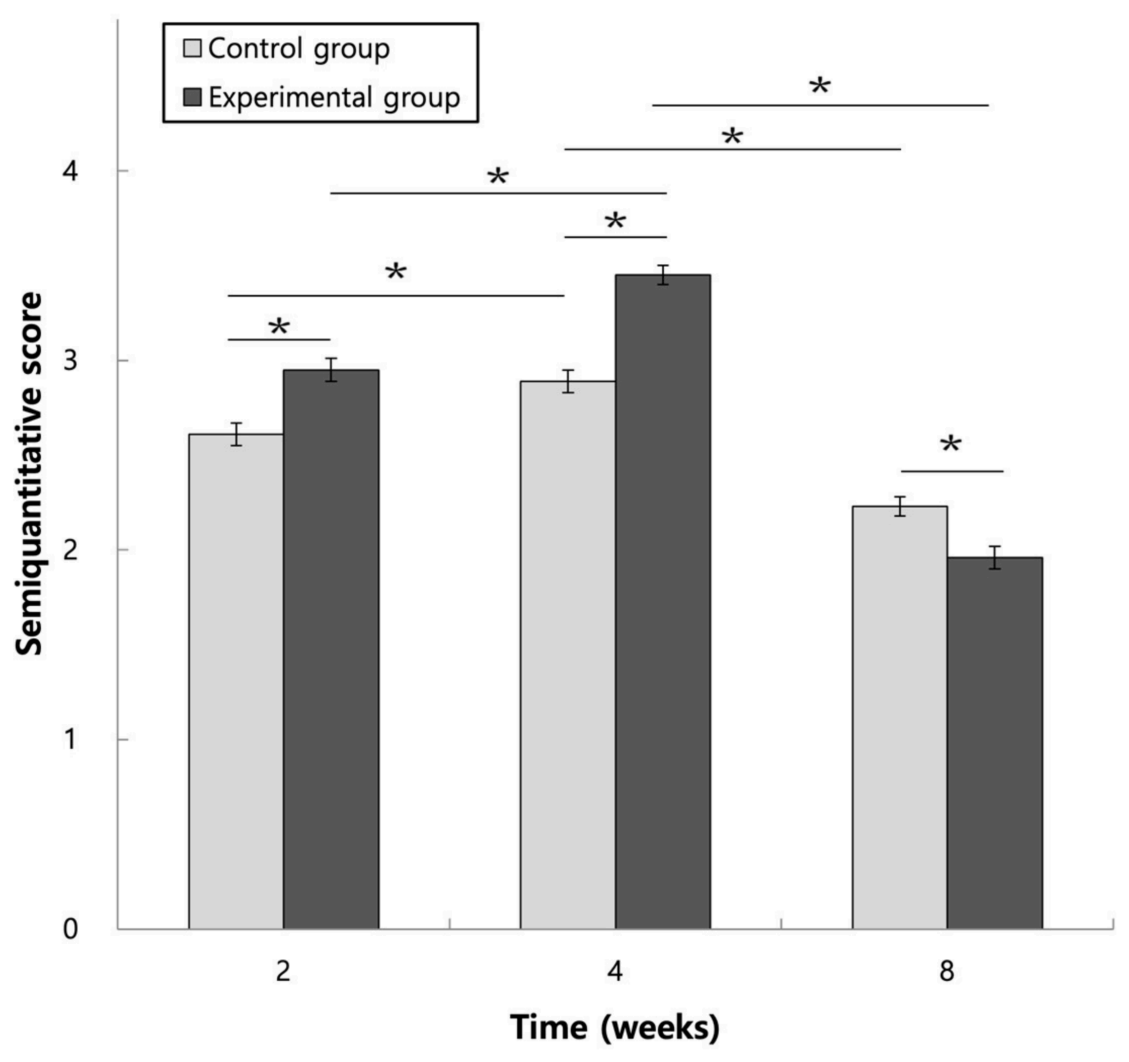
Semiquantitative scoring of PCNA immunostaining in the control and experimental groups at 2, 4, and 8 weeks. (* *p* < 0.05).

Semiquantitative scores for the control group were 2.61 ± 0.06, 2.89 ± 0.06, and 2.23 ± 0.05 at 2, 4, and 8 weeks, respectively, compared with 2.95 ± 0.06, 3.45 ± 0.05, and 1.96 ± 0.06 in the experimental group. PCNA expression reached its highest level at 4 weeks in both groups and was significantly greater at this time point than at 2 and 8 weeks (*p* < 0.05). Compared with the control group, the experimental group showed significantly higher PCNA expression at 2 and 4 weeks but significantly lower expression at 8 weeks (*p* < 0.05) (Figure 3).

### 3.2. Expression of VEGF

**Figure 4:**
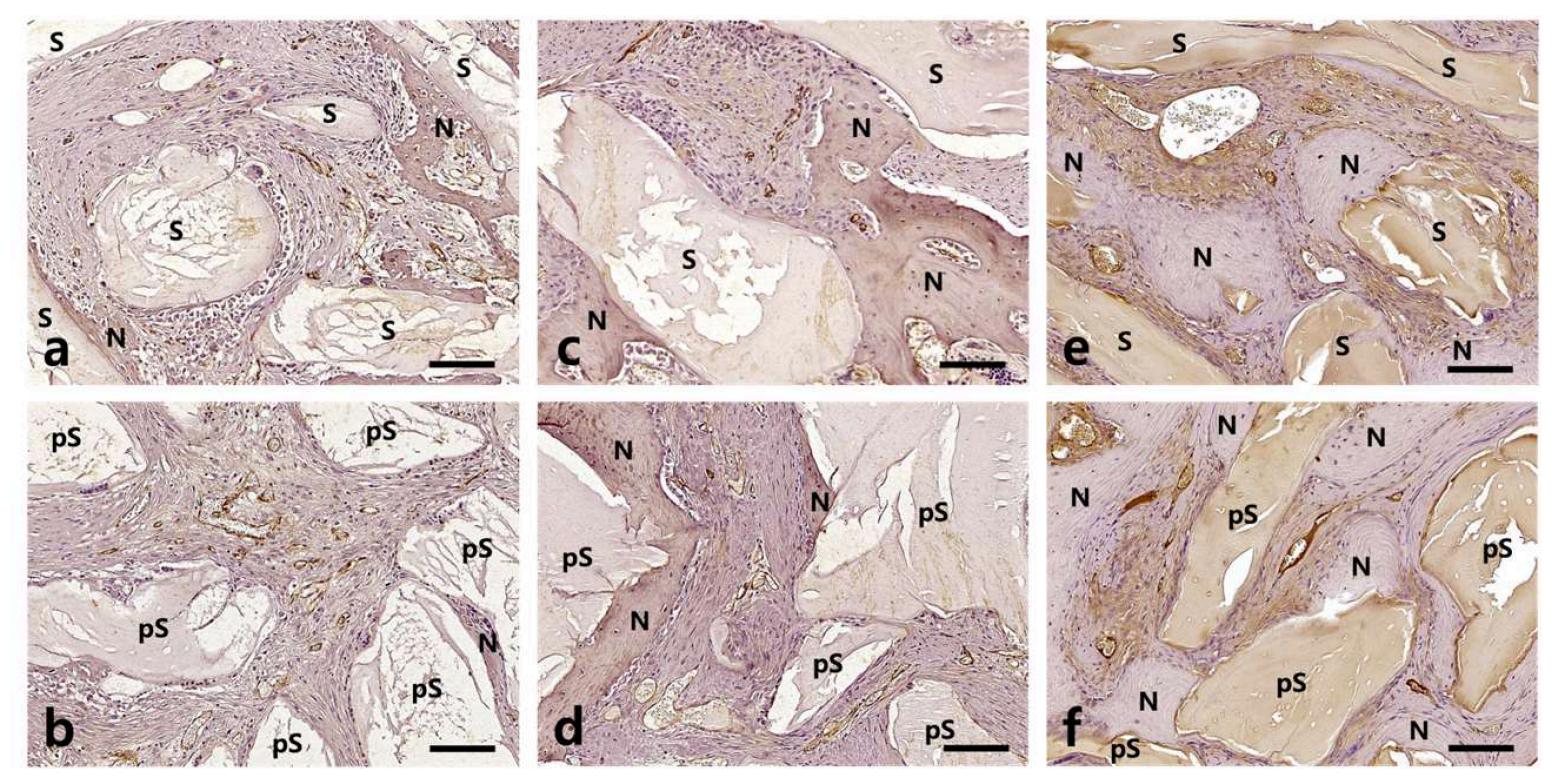
Immunohistochemical staining for VEGF in control (**a**,**c**,**e**) and experimental (**b**,**d**,**f**) groups at 2 (**a**,**b**), 4 (**c**,**d**), and 8 (**e**,**f**) weeks after surgery. Abbreviations: N, newly formed bone; S, SANTA-OSS particles; pS, plasma-treated SANTA-OSS particles (Scale bar: 100 μm).

In the control specimens, at 2 weeks after surgery, numerous VEGF-positive cells were detected on the surfaces of both newly formed bone and the SANTA-OSS particles and within the surrounding connective tissue (Figure 4a). At 4 and 8 weeks after surgery, VEGF expression decreased, with some VEGF-positive cells detected in capillaries within the surrounding connective tissue (Figure 4c,e). In the experimental specimens, at 2 weeks after surgery, strong VEGF expression was detected along the surfaces of the newly formed bone and the plasma-treated SANTA-OSS particles and within the surrounding connective tissue. VEGF-positive cells were rarely observed on the capillaries in the surrounding connective tissue (Figure 4b). At 4 weeks after surgery, VEGF expression decreased, with numerous VEGF-positive cells detected along the newly formed bone surface and on the capillaries in the surrounding connective tissue (Figure 4d). At 8 weeks, VEGF expression decreased, with some VEGF-positive cells detected on the capillaries in the surrounding connective tissue (Figure 4f).

**Figure 5:**
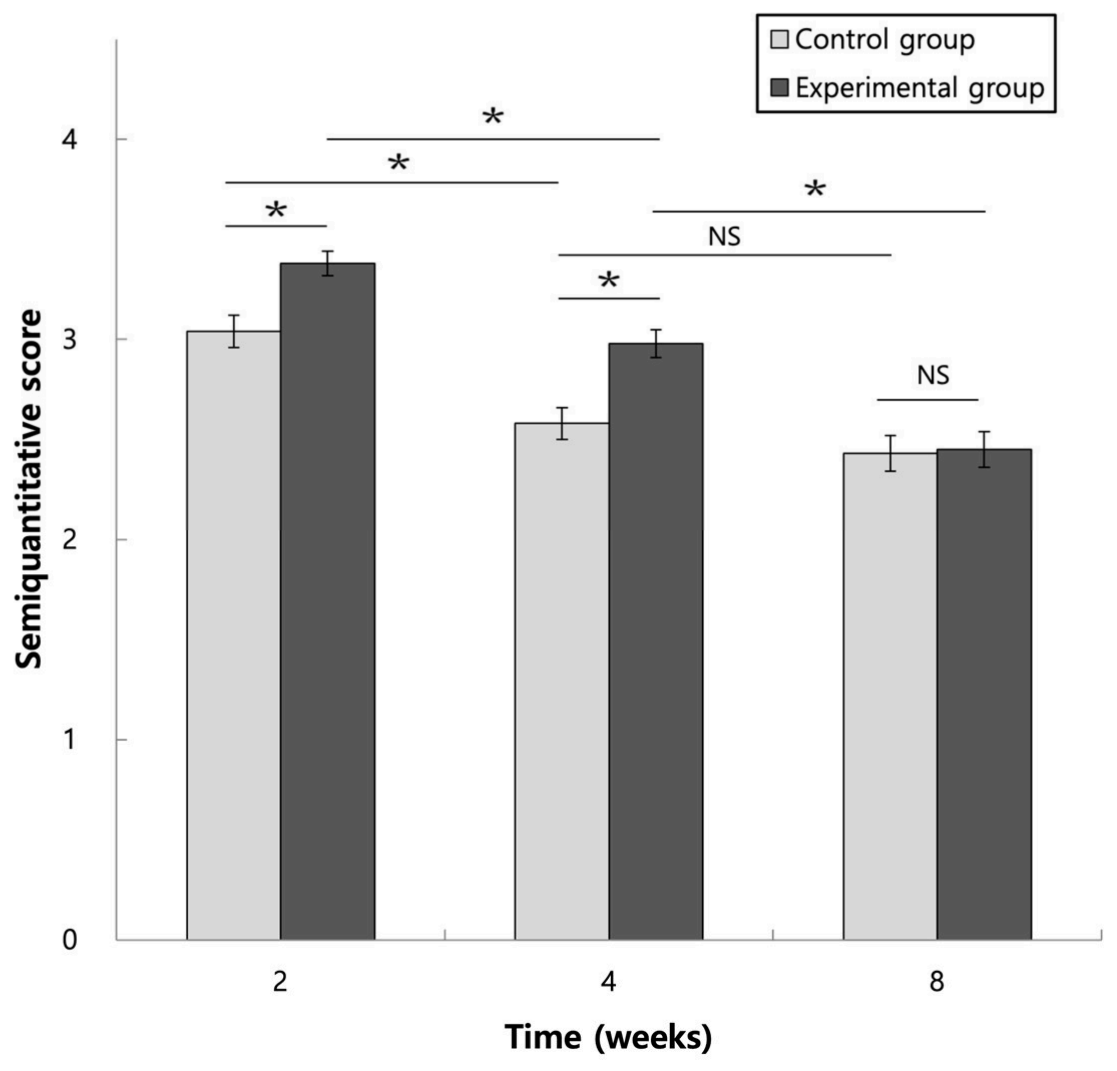
Semiquantitative scoring of VEGF immunostaining in the control and experimental groups at 2, 4, and 8 weeks. (* *p* < 0.05).

In the control group, semiquantitative VEGF expression scores at 2, 4, and 8 weeks were 3.04 ± 0.08, 2.58 ± 0.08, and 2.43 ± 0.09, respectively. For the experimental group, VEGF scores at the same time points were 3.38 ± 0.06, 2.98 ± 0.07, and 2.45 ± 0.09, respectively. At 2 weeks, VEGF expression was significantly higher than at 4 and 8 weeks in both groups (*p* < 0.05). In the experimental group, VEGF expression increased significantly at 2 and 4 weeks compared with the control group. At 8 weeks, VEGF expression in the experimental group was not significantly different from that in the control group (Figure 5).

### 3.3. Expression of PECAM-1

**Figure 6:**
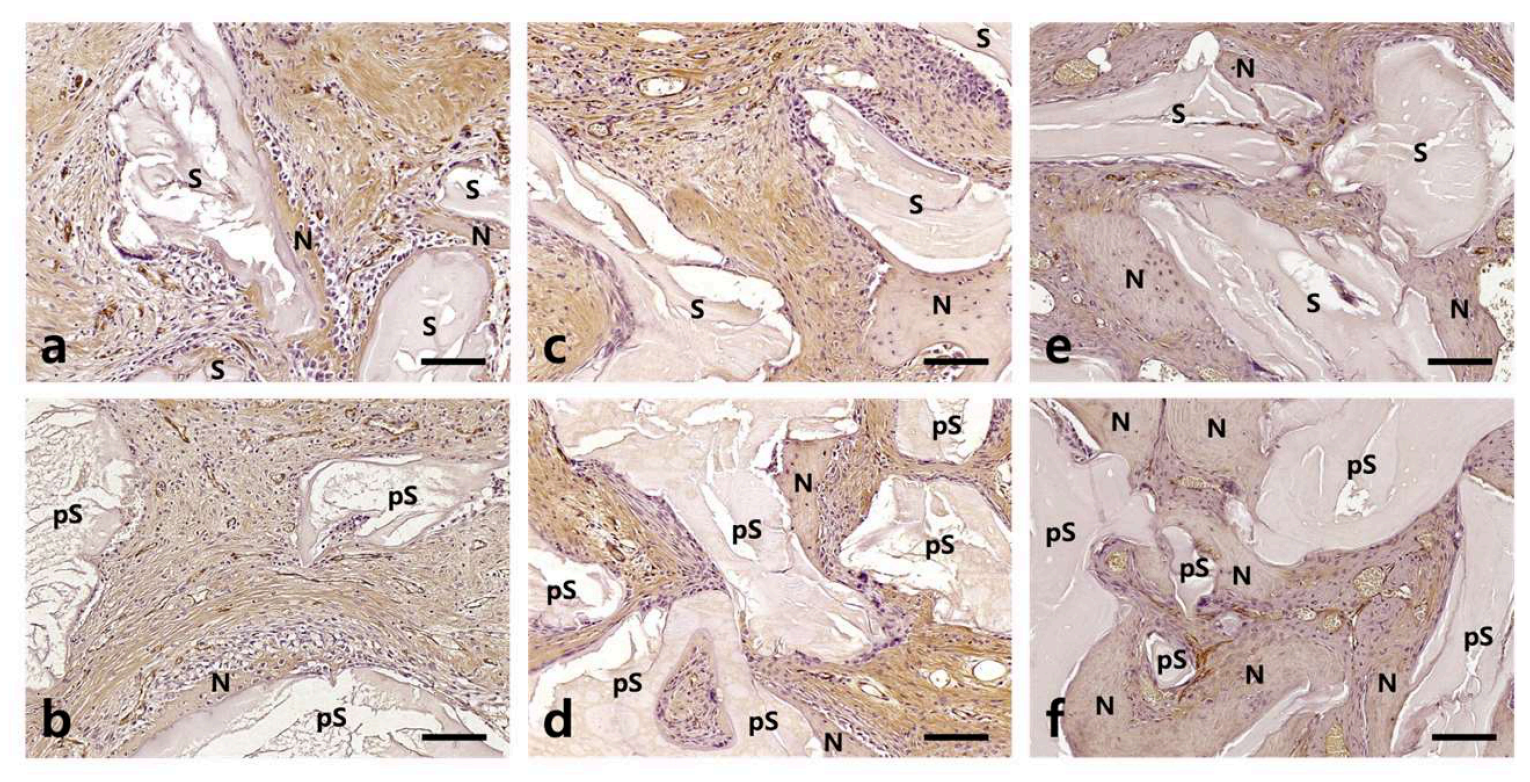
Immunohistochemical staining for PECAM-1 in control (**a**,**c**,**e**) and experimental (**b**,**d**,**f**) groups at 2 (**a**,**b**), 4 (**c**,**d**), and 8 (**e**,**f**) weeks after surgery. Abbreviations: N, newly formed bone; S, SANTA-OSS particles; pS, plasma-treated SANTA-OSS particles (Scale bar: 100 μm).

In the control specimens, at 2 weeks after surgery, numerous PECAM-1-positive cells were detected on the surface of the SANTA-OSS particles and within the surrounding connective tissue (Figure 6a). At 4 and 8 weeks after surgery, PECAM-1 expression decreased, with some PECAM-1-positive cells detected along the newly formed bone surface and on capillaries within the surrounding connective tissue (Figure 6c,e). In the experimental specimens, at 2 weeks after surgery, strong PECAM-1 expression was detected along the surfaces of the newly formed bone and the plasma-treated SANTA-OSS particles and within the surrounding connective tissue. PECAM-1-positive cells were rarely observed on the capillaries in the surrounding connective tissue (Figure 6b). At 4 weeks after surgery, PECAM-1 expression decreased, with numerous PECAM-1-positive cells detected along the surfaces of the newly formed bone and plasma-treated SANTA-OSS particles, as well as on capillaries within the surrounding connective tissue (Figure 6d). At 8 weeks, some PECAM-1-positive cells were detected on the capillaries within the surrounding connective tissue (Figure 6f).

**Figure 7:**
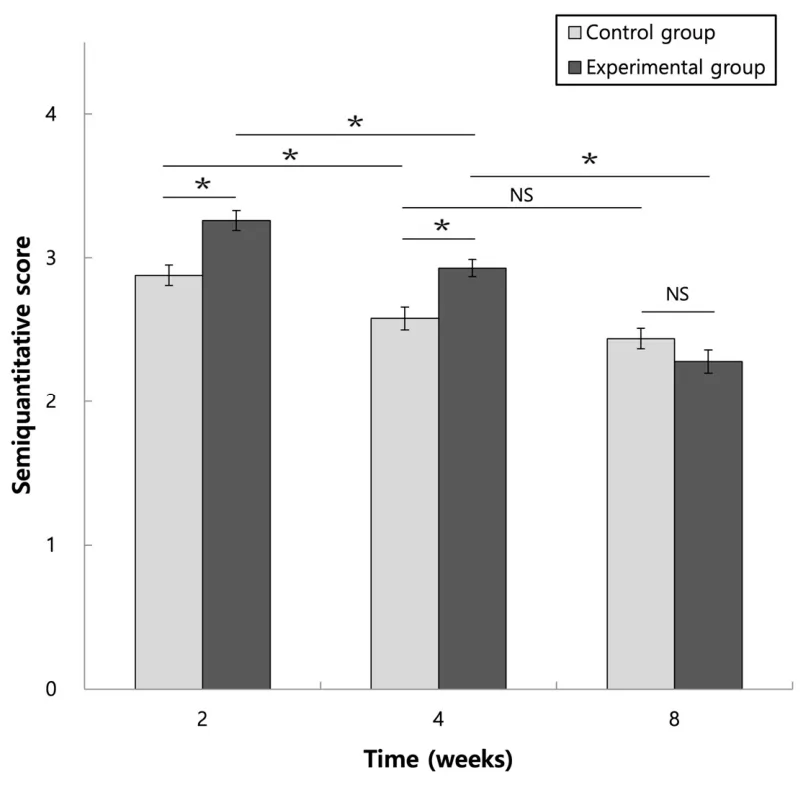
Semiquantitative scoring of PECAM-1 immunostaining in the control and experimental groups at 2, 4, and 8 weeks. (* *p* < 0.05).

In the control group, the semiquantitative PECAM-1 expression scores at 2, 4, and 8 weeks were 2.88 ± 0.07, 2.58 ± 0.08, and 2.44 ± 0.07, respectively. For the experimental group, PECAM-1 scores at the same time points were 3.26 ± 0.07, 2.93 ± 0.06, and 2.28 ± 0.08, respectively. At 2 weeks, PECAM-1 expression was significantly greater than at 4 and 8 weeks in both groups (*p* < 0.05). In the experimental group, PECAM-1 expression increased significantly at 2 and 4 weeks compared with the control group. At 8 weeks, PECAM-1 expression in the experimental group was not significantly different from that in the control group (Figure 7).

### 3.4. Expression of Collagen I

**Figure 8:**
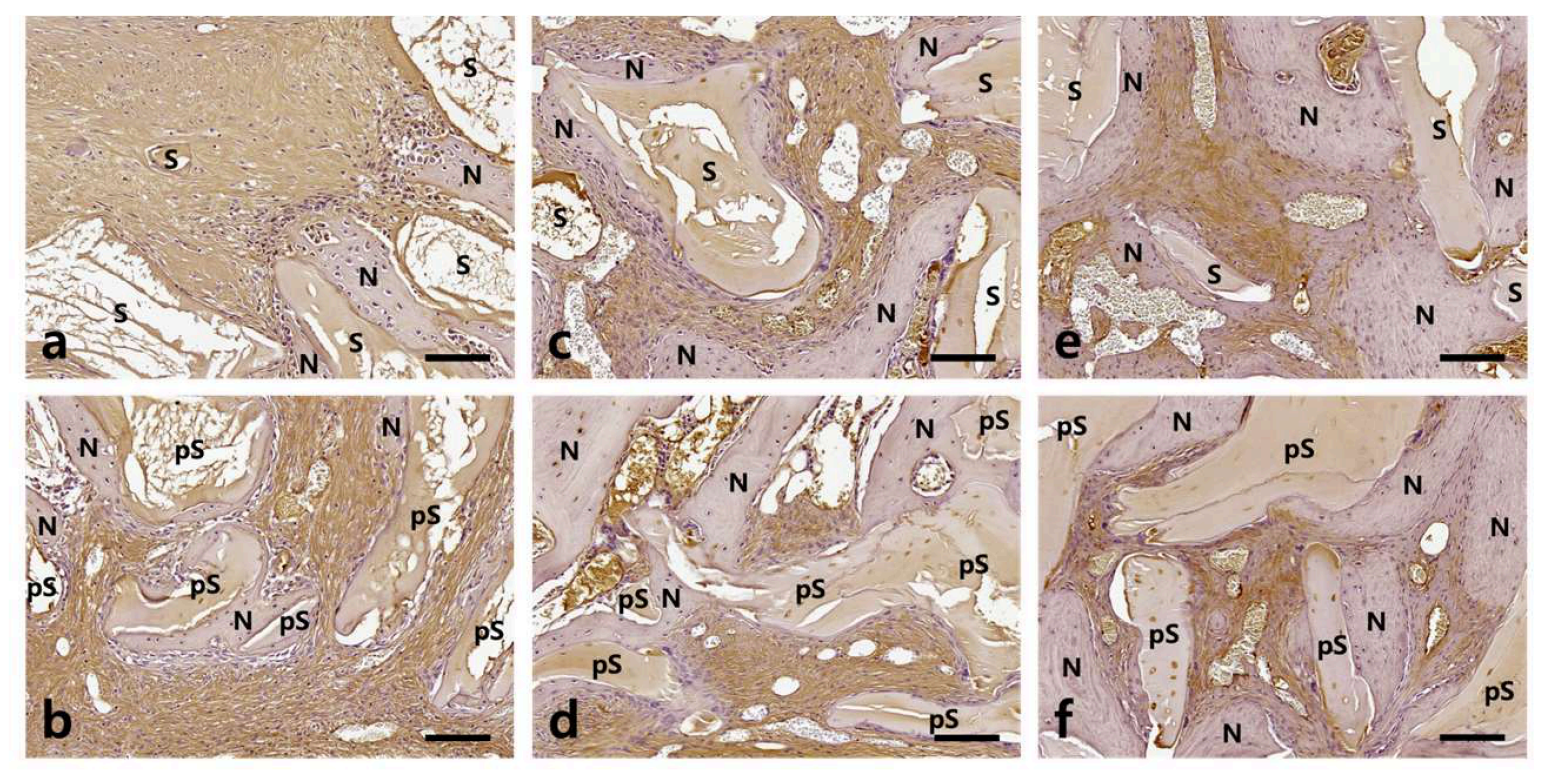
Immunohistochemical staining for collagen I in control (**a**,**c**,**e**) and experimental (**b**,**d**,**f**) groups at 2 (**a**,**b**), 4 (**c**,**d**), and 8 (**e**,**f**) weeks after surgery. Abbreviations: N, newly formed bone; S, SANTA-OSS particles; pS, plasma-treated SANTA-OSS particles (Scale bar: 100 μm).

In the control specimens, at 2 weeks after surgery, moderate collagen I expression was detected on the surfaces of the newly formed bone and the SANTA-OSS particles and in osteoblasts (Figure 8a). At 4 weeks after surgery, the control specimens showed mild collagen I expression on the surface of the SANTA-OSS particles and within the surrounding connective tissue (Figure 8c). At 8 weeks after surgery, the control specimens showed weak collagen I expression on the surface of the SANTA-OSS particles and within the surrounding connective tissue (Figure 8e). In the experimental specimens, at 2 weeks after surgery, strong collagen I expression was detected on the surfaces of the newly formed bone and the plasma-treated SANTA-OSS particles and within the surrounding connective tissue (Figure 8b). At 4 weeks after surgery, collagen I expression decreased, with moderate expression detected on the surfaces of the newly formed bone and plasma-treated SANTA-OSS particles, as well as within the surrounding connective tissue (Figure 8d). At 8 weeks after surgery, the experimental specimens showed mild collagen I expression on the surface of the newly formed bone and within the surrounding connective tissue (Figure 8f).

**Figure 9:**
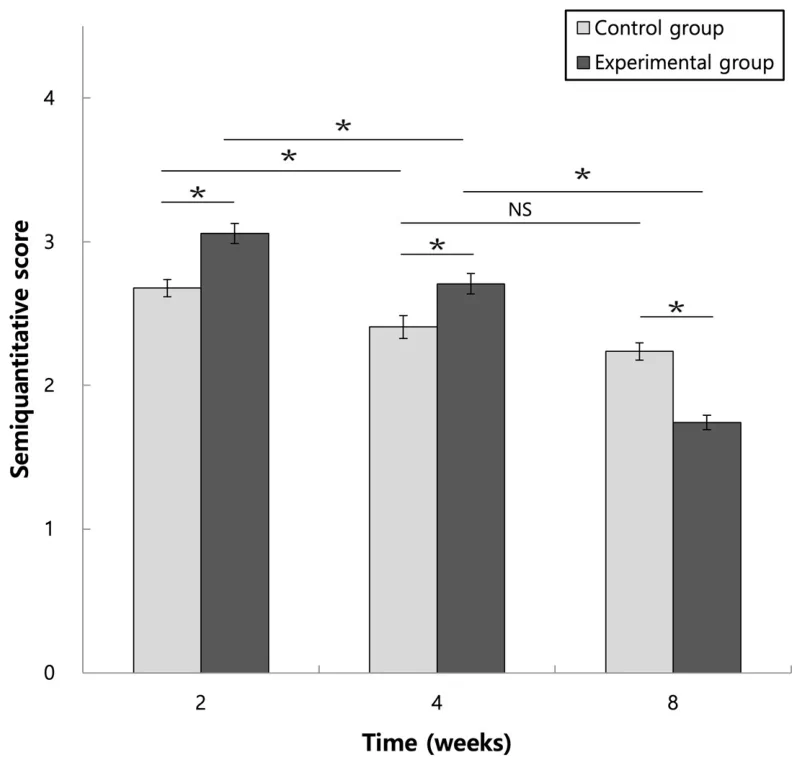
Semiquantitative scoring of collagen I immunostaining in the control and experimental groups at 2, 4, and 8 weeks. (* *p* < 0.05).

In the control group, the semiquantitative collagen I expression scores at 2, 4, and 8 weeks were 2.68 ± 0.06, 2.41 ± 0.08, and 2.24 ± 0.06, respectively. For the experimental group, collagen I scores at the same time points were 3.06 ± 0.07, 2.71 ± 0.07, and 1.74 ± 0.05, respectively. At 2 weeks, collagen I expression was significantly higher than at 4 and 8 weeks in both groups (*p* < 0.05). In the experimental group, collagen I expression increased significantly at 2 and 4 weeks compared with the control group. At 8 weeks, the experimental group showed significantly lower collagen I expression than in the control group (*p* < 0.05) (Figure 9).

### 3.5. Expression of Collagen III

**Figure 10:**
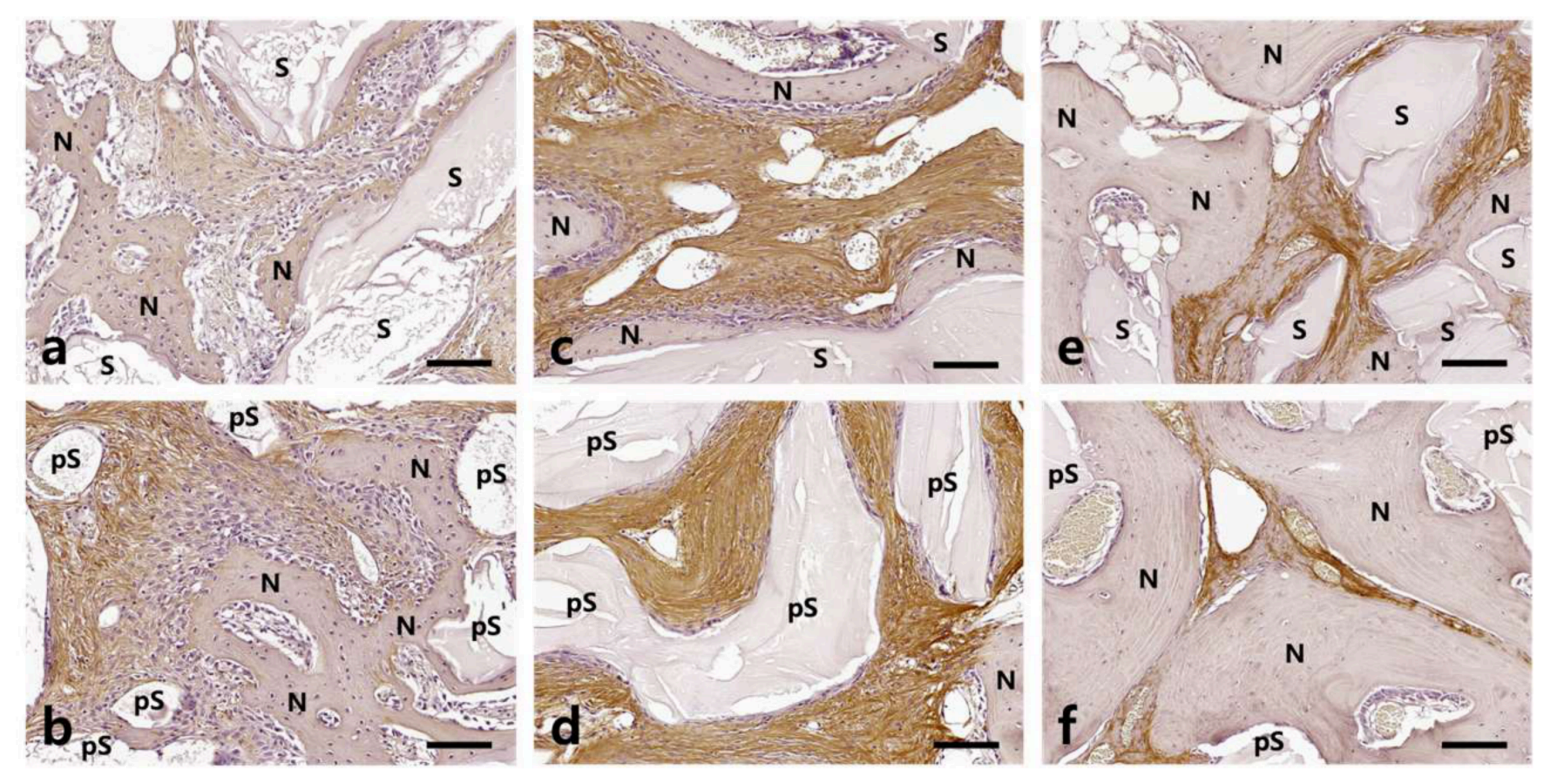
Immunohistochemical staining for collagen in control (**a**,**c**,**e**) and experimental (**b**,**d**,**f**) groups at 2 (**a**,**b**), 4 (**c**,**d**), and 8 (**e**,**f**) weeks after surgery. Abbreviations: N, newly formed bone; S, SANTA-OSS particles; pS, plasma-treated SANTA-OSS particles (Scale bar: 100 μm).

In the control specimens, at 2 weeks after surgery, weak collagen III expression was detected on the surfaces of the newly formed bone and on the SANTA-OSS particles and within the surrounding connective tissue (Figure 10a). At 4 and 8 weeks after surgery, collagen III expression increased, with moderate expression detected along the surfaces of the newly formed bone and the SANTA-OSS particles and within the surrounding connective tissue (Figure 10c,e). In the experimental specimens, at 2 weeks after surgery, moderate collagen III expression was detected along the surfaces of the newly formed bone and the plasma-treated SANTA-OSS particles and within the surrounding connective tissue (Figure 10b). At 4 weeks after surgery, collagen III expression increased, with strong expression detected along the surface of the newly formed bone and the plasma-treated SANTA-OSS particles and within the surrounding connective tissue (Figure 10d). At 8 weeks after surgery, collagen III expression decreased, with mild expression detected along the newly formed bone surface and within the surrounding connective tissue (Figure 10f).

**Figure 11:**
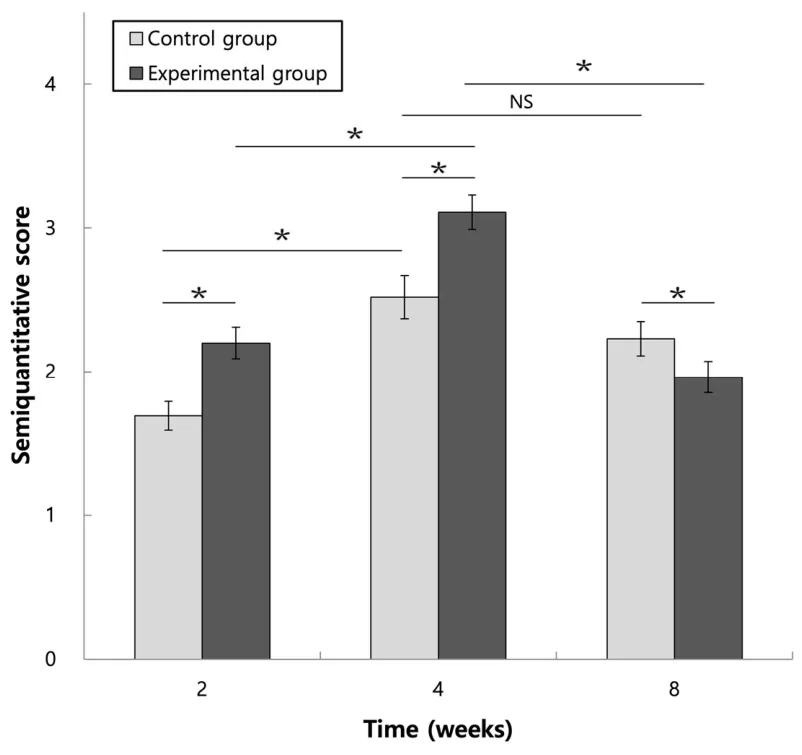
Semiquantitative scoring of collagen III immunostaining in the control and experimental groups at 2, 4, and 8 weeks. (* *p* < 0.05).

In the control group, the semiquantitative collagen III expression scores at 2, 4, and 8 weeks were 1.69 ± 0.10, 2.52 ± 0.15, and 2.23 ± 0.12, respectively. For the experimental group, collagen III scores at the same time points were 2.20 ± 0.11, 3.11 ± 0.12, and 1.96 ± 0.11, respectively. At 4 weeks, collagen III expression was significantly higher than at 2 and 8 weeks in both groups (*p* < 0.05). In the experimental group, collagen III expression increased significantly at 2 and 4 weeks compared with the control group. At 8 weeks, the experimental group showed significantly lower collagen III expression than in the control group (*p* < 0.05) (Figure 11).

### 3.6. Expression of ALP

**Figure 12:**
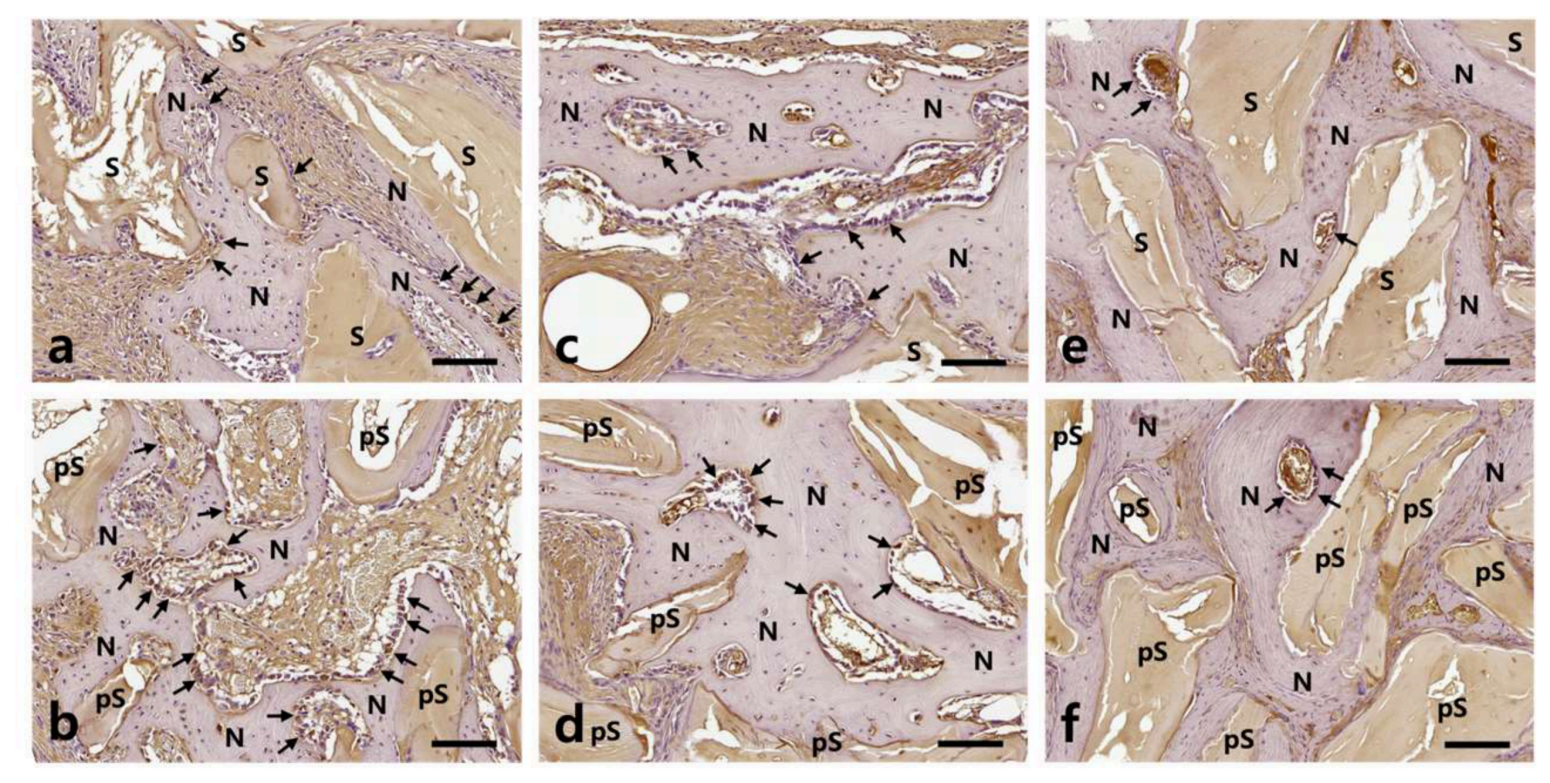
Immunohistochemical staining for ALP in control (**a**,**c**,**e**) and experimental (**b**,**d**,**f**) groups at 2 (**a**,**b**), 4 (**c**,**d**), and 8 (**e**,**f**) weeks after surgery. Arrows indicate ALP-positive cells. Abbreviations: N, newly formed bone; S, SANTA-OSS particles; pS, plasma-treated SANTA-OSS particles (Scale bar: 100 μm).

In the control specimens, at 2 weeks after surgery, many ALP-positive osteoblasts were detected along the surfaces of the newly formed bone and the SANTA-OSS particles (Figure 12a). At 4 and 8 weeks after surgery, ALP expression decreased, with some ALP-positive osteoblasts detected along the newly formed bone surface (Figure 12c,e). In the experimental specimens, at 2 weeks after surgery, numerous ALP-positive osteoblasts were detected along the newly formed bone surface (Figure 12b). At 4 weeks after surgery, ALP expression decreased, with some ALP-positive osteoblasts detected along the newly formed bone surface (Figure 12d). At 8 weeks after surgery, some ALP-positive osteoblasts were detected around the newly formed bone surface (Figure 12f).

**Figure 13:**
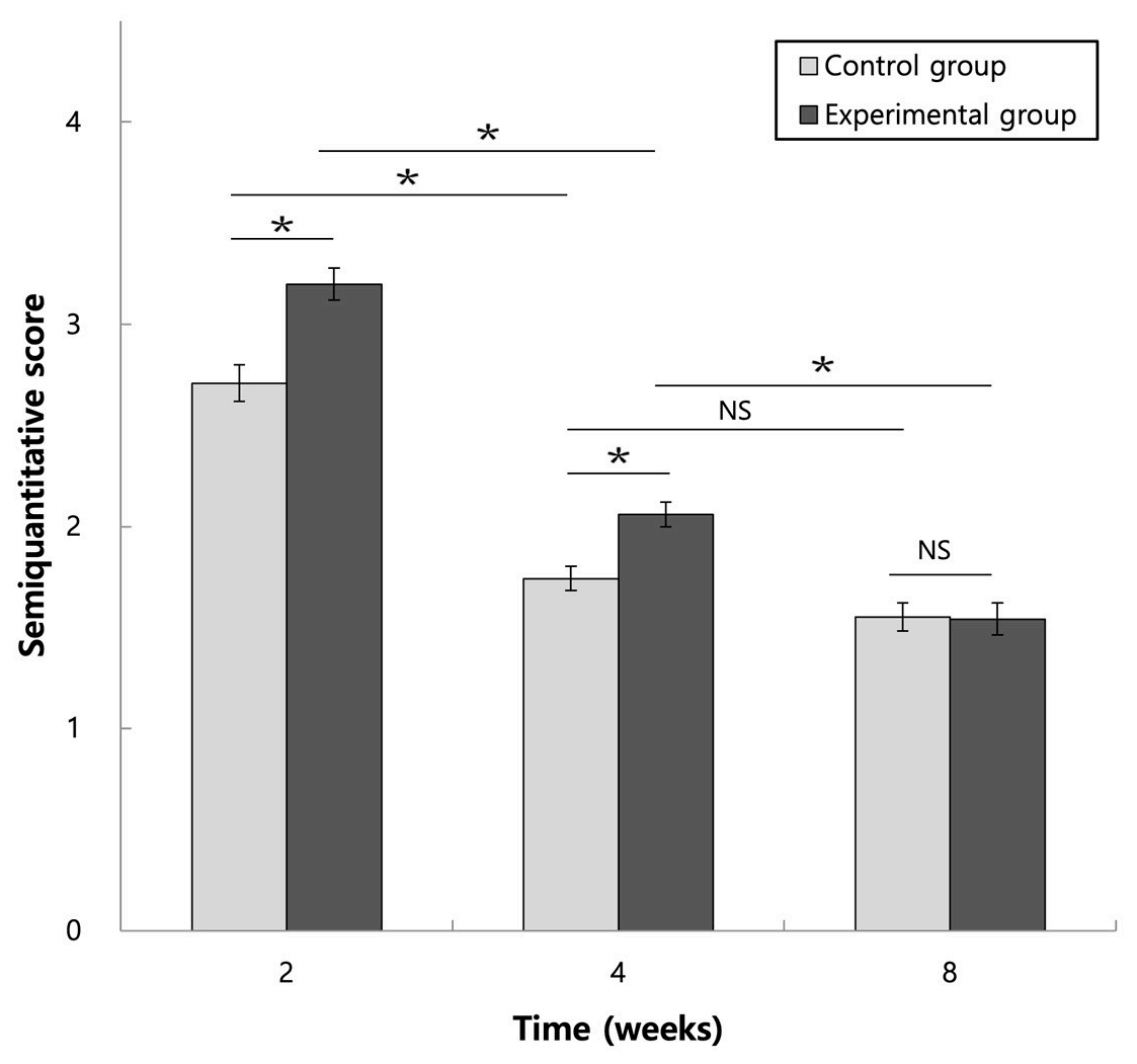
Semiquantitative scoring of ALP immunostaining in the control and experimental groups at 2, 4, and 8 weeks. (* *p* < 0.05).

In the control group, the semiquantitative ALP expression scores at 2, 4, and 8 weeks were 2.71 ± 0.09, 1.74 ± 0.06, and 1.55 ± 0.07, respectively. For the experimental group, ALP scores at the same time points were 3.20 ± 0.08, 2.06 ± 0.06, and 1.54 ± 0.08, respectively. At 2 weeks, ALP expression was significantly higher than at 4 and 8 weeks in both groups (*p* < 0.05). The experimental group showed significantly higher ALP expression than the control group at 2 and 4 weeks. At 8 weeks, ALP expression did not differ significantly between the experimental and control groups (Figure 13).

### 3.7. Expression of BMP2

**Figure 14:**
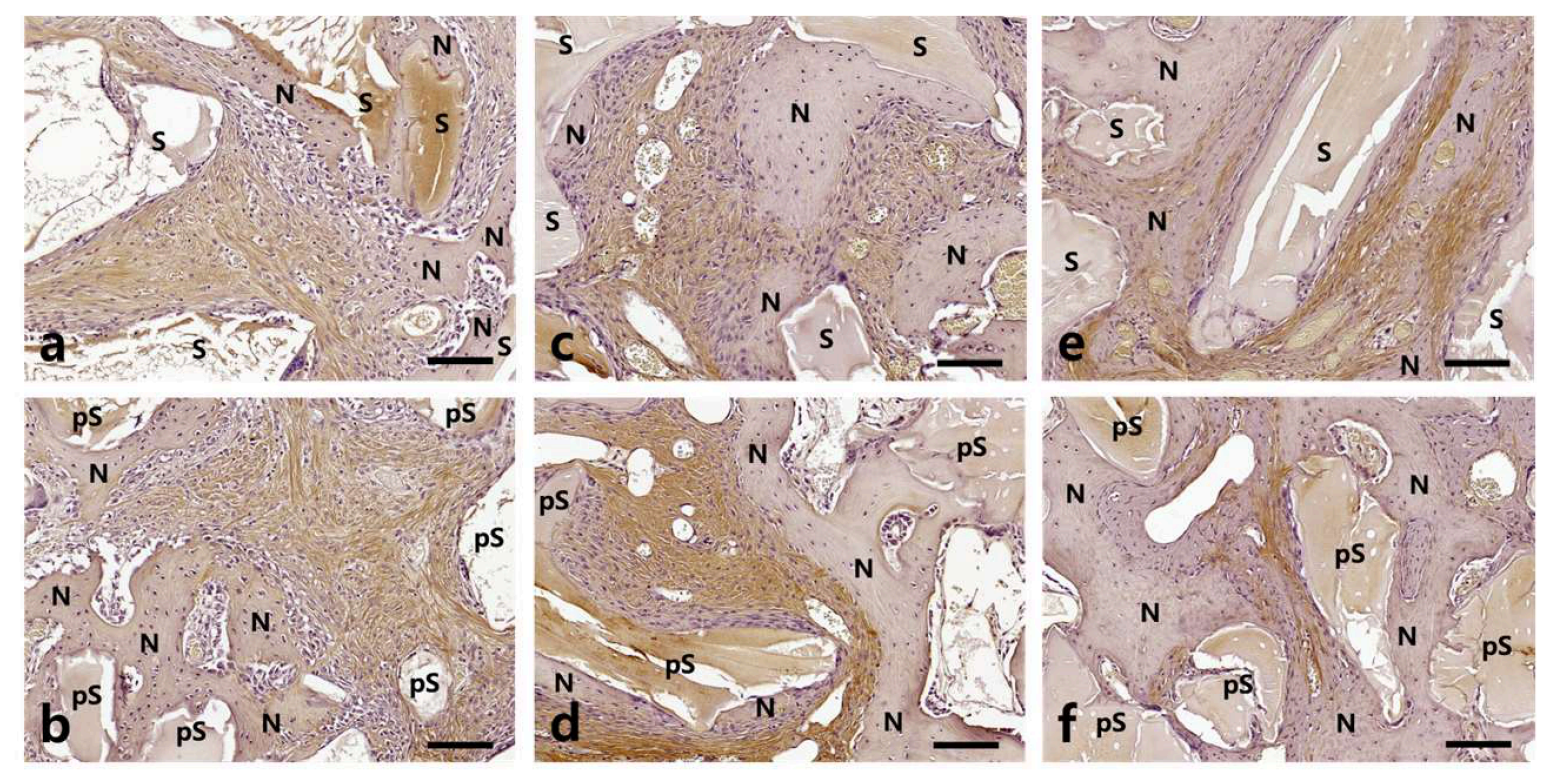
Immunohistochemical staining for BMP2 in control (**a**,**c**,**e**) and experimental (**b**,**d**,**f**) groups at 2 (**a**,**b**), 4 (**c**,**d**), and 8 (**e**,**f**) weeks after surgery. Abbreviations: N, newly formed bone; S, SANTA-OSS particles; pS, plasma-treated SANTA-OSS particles (Scale bar: 100 μm).

In the control specimens, at 2 weeks after surgery, weak BMP2 was detected along the surfaces of the newly formed bone and the SANTA-OSS particles and within the surrounding connective tissue (Figure 14a). At 4 weeks after surgery, BMP2 expression increased, with moderate expression detected along the surfaces of the newly formed bone and the SANTA-OSS particles and within the surrounding connective tissue (Figure 14c). At 8 weeks after surgery, BMP2 expression decreased, with mild expression detected along the surfaces of the newly formed bone and the SANTA-OSS particles and within the surrounding connective tissue (Figure 14e). In the experimental specimens, at 2 weeks after surgery, moderate BMP2 expression was detected along the surfaces of the newly formed bone and the plasma-treated SANTA-OSS particles and within the surrounding connective tissue (Figure 14b). At 4 weeks after surgery, BMP2 expression increased, with strong expression detected along the surface of the newly formed bone and the plasma-treated SANTA-OSS particles and within fibroblast-rich dense connective tissue (Figure 14d). At 8 weeks after surgery, BMP2 expression decreased, with weak expression detected along the newly formed bone surface and within the surrounding connective tissue (Figure 14f).

**Figure 15:**
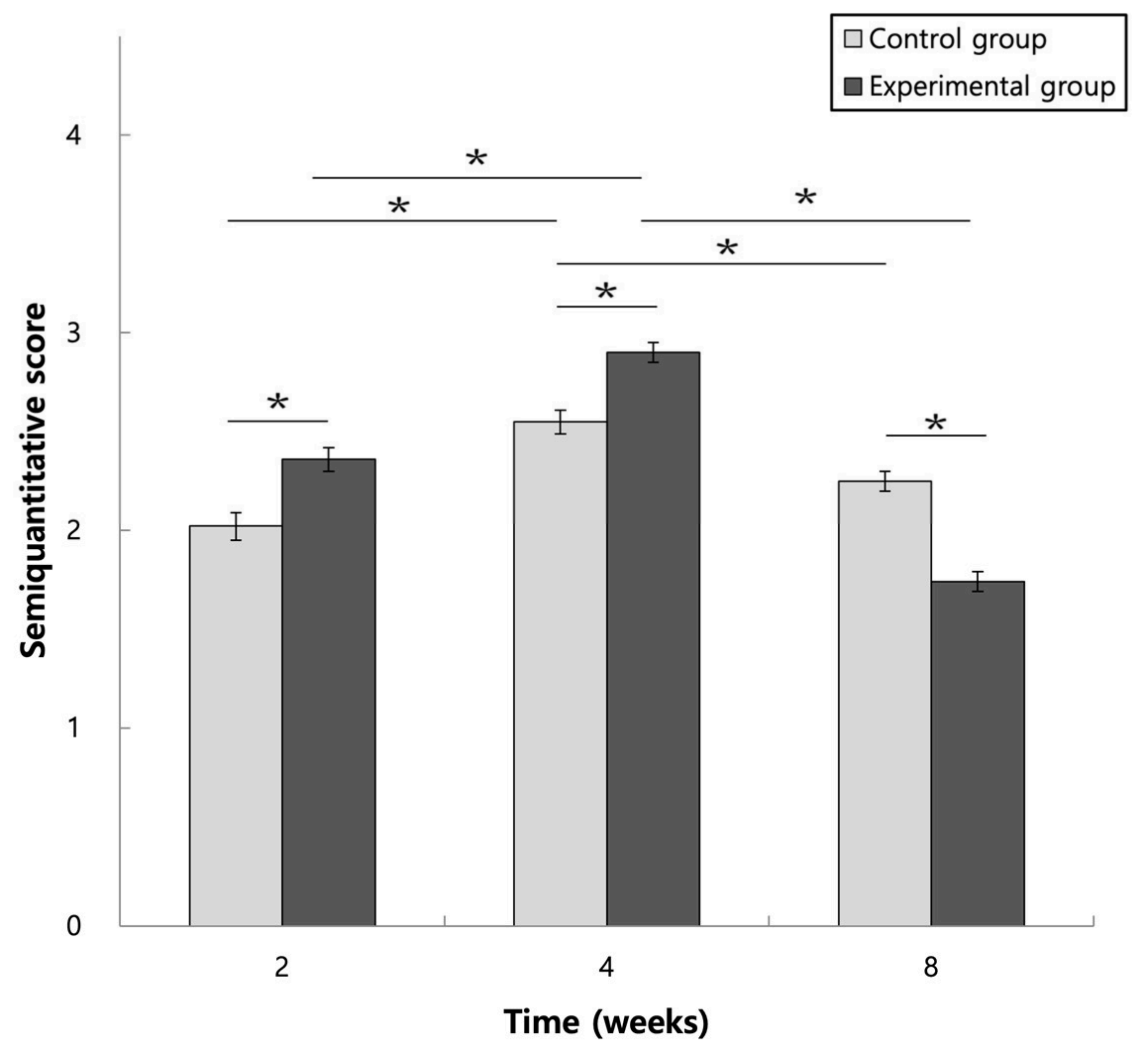
Semiquantitative scoring of BMP2 immunostaining in the control and experimental groups at 2, 4, and 8 weeks. (* *p* < 0.05).

In the control group, the semiquantitative BMP2 expression scores at 2, 4, and 8 weeks were 2.02 ± 0.07, 2.55 ± 0.06, and 2.25 ± 0.05, respectively. For the experimental group, BMP2 scores at the same time points were 2.36 ± 0.06, 2.90 ± 0.05, and 1.74 ± 0.05, respectively. At 4 weeks, BMP2 expression was significantly higher than at 2 and 8 weeks in both groups (*p* < 0.05). The experimental group showed significantly higher BMP2 expression than the control group at 2 and 4 weeks (*p* < 0.05). At 8 weeks, the experimental group showed significantly lower BMP2 expression than the control group (*p* < 0.05) (Figure 15).

### 3.8. Expression of BMP4

**Figure 16:**
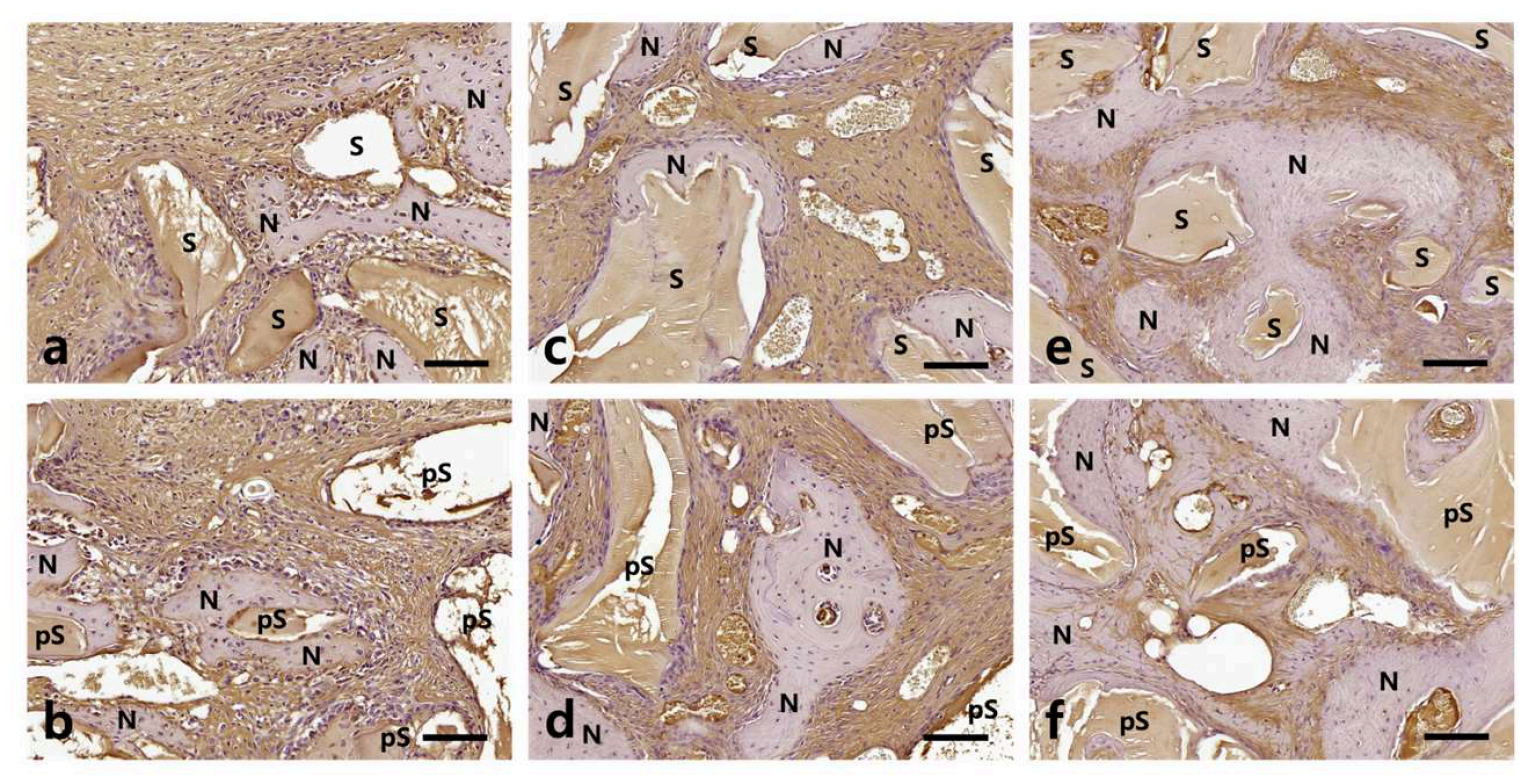
Immunohistochemical staining for BMP4 in control (**a**,**c**,**e**) and experimental (**b**,**d**,**f**) groups at 2 (**a**,**b**), 4 (**c**,**d**), and 8 (**e**,**f**) weeks after surgery. Abbreviations: N, newly formed bone; S, SANTA-OSS particles; pS, plasma-treated SANTA-OSS particles (Scale bar: 100 μm).

In the control specimens, at 2 and 4 weeks after surgery, moderate BMP4 expression was detected along the surfaces of the newly formed bone and the SANTA-OSS particles and within the surrounding connective tissue (Figure 16a,c). At 8 weeks after surgery, BMP4 expression decreased, with mild expression detected along the surface of the newly formed bone and the SANTA-OSS particles and within the surrounding connective tissue (Figure 16e). In the experimental specimens, at 2 and 4 weeks after surgery, strong BMP4 expression was detected along the surfaces of the newly formed bone and the plasma-treated SANTA-OSS particles and within the surrounding connective tissue (Figure 16b,d). At 8 weeks after surgery, BMP4 expression decreased, with weak expression detected along the surfaces of the newly formed bone and the plasma-treated SANTA-OSS particles and within the surrounding connective tissue (Figure 16f).

**Figure 17:**
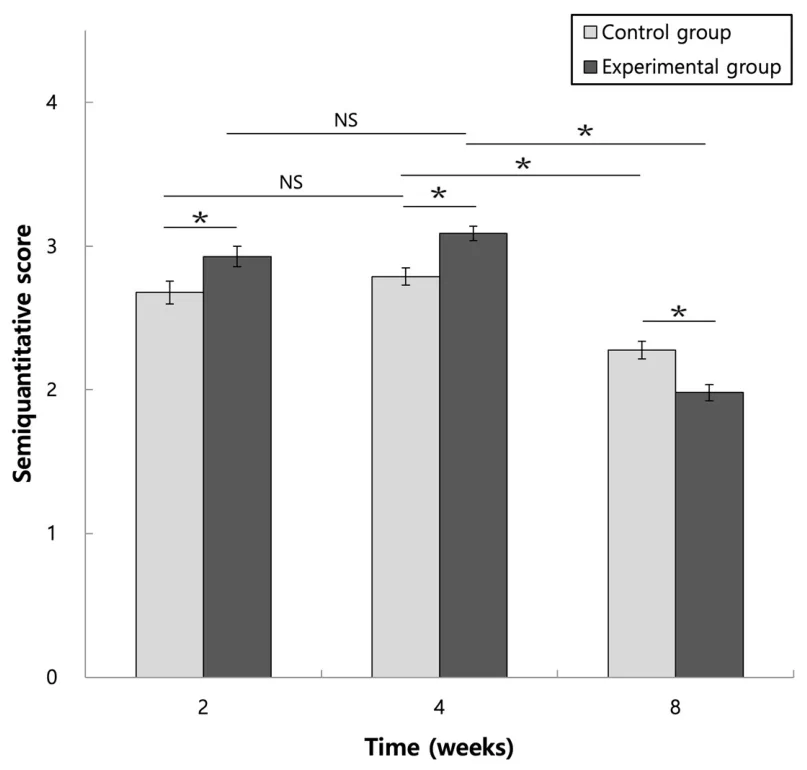
Semiquantitative scoring of BMP4 immunostaining in the control and experimental groups at 2, 4, and 8 weeks. (* *p* < 0.05).

In the control group, the semiquantitative BMP4 expression scores at 2, 4, and 8 weeks were 2.68 ± 0.08, 2.79 ± 0.06, and 2.28 ± 0.06, respectively. For the experimental group, BMP4 scores at the same time points were 2.93 ± 0.07, 3.09 ± 0.05, and 1.98 ± 0.06, respectively. At 2 and 4 weeks, BMP4 expression was significantly higher than at 8 weeks in both groups (*p* < 0.05). The experimental group showed significantly higher BMP4 expression than the control group at 2 and 4 weeks (*p* < 0.05). At 8 weeks, the experimental group showed significantly lower BMP4 expression than the control group (Figure 17).

### 3.9. Expression of BMP7

**Figure 18:**
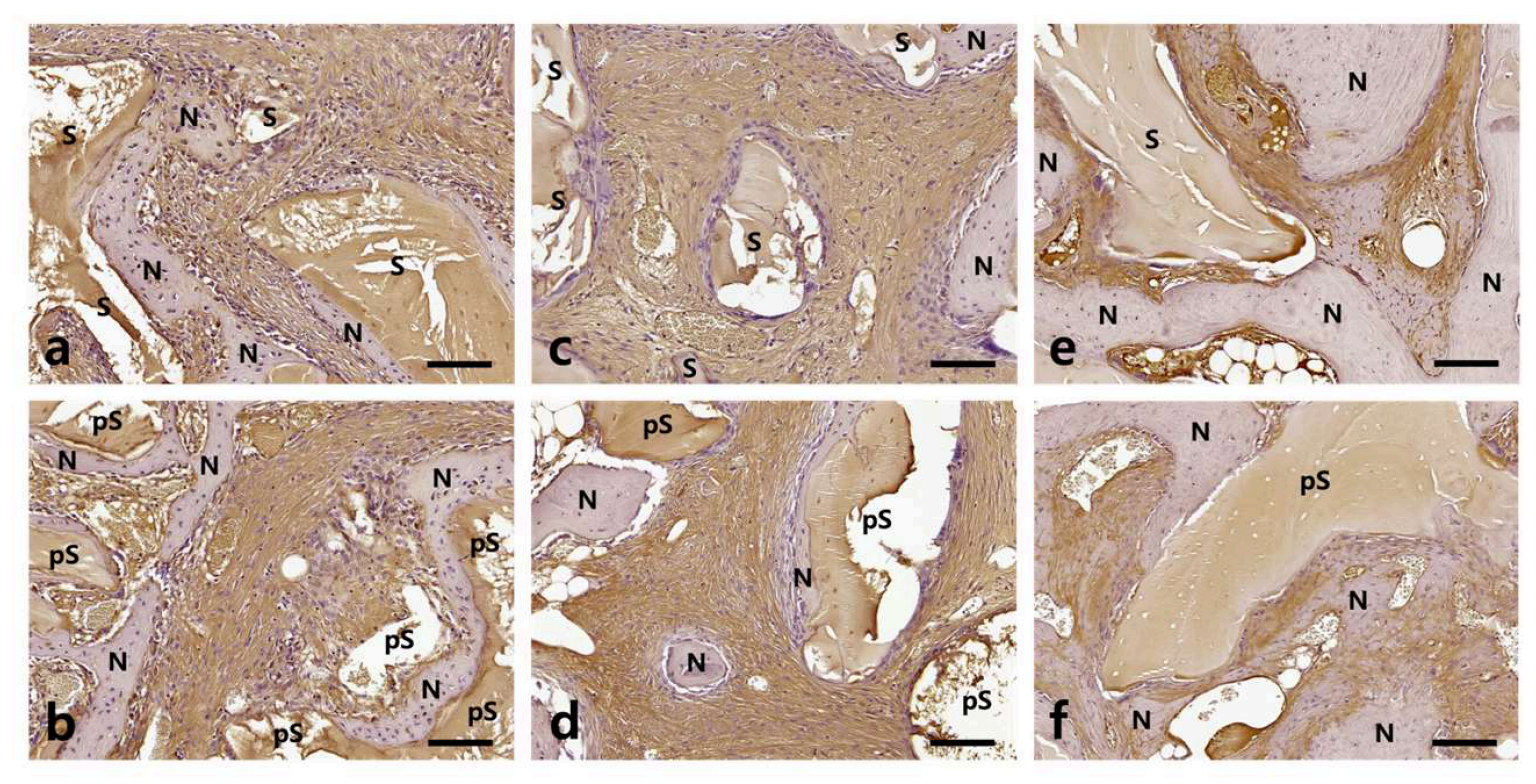
Immunohistochemical staining for BMP7 in control (**a**,**c**,**e**) and experimental (**b**,**d**,**f**) groups at 2 (**a**,**b**), 4 (**c**,**d**), and 8 (**e**,**f**) weeks after surgery. Abbreviations: N, newly formed bone; S, SANTA-OSS particles; pS, plasma-treated SANTA-OSS particles (Scale bar: 100 μm).

In the control specimens, at 2 and 4 weeks after surgery, moderate BMP7 expression was detected along the surfaces of the newly formed bone and on SANTA-OSS particles and within the surrounding connective tissue (Figure 18a,c). At 8 weeks after surgery, BMP7 expression decreased, with mild expression detected along the surfaces of the newly formed bone and the SANTA-OSS particles and within the surrounding connective tissue (Figure 18e). In the experimental specimens, at 2 and 4 weeks after surgery, strong BMP7 expression was detected along the surfaces of the newly formed bone and the plasma-treated SANTA-OSS particles and within the surrounding connective tissue (Figure 18b,d). At 8 weeks, BMP7 expression decreased, with weak expression detected along the surfaces of the newly formed bone and the plasma-treated SANTA-OSS particles and within the surrounding connective tissue (Figure 18f).

**Figure 19:**
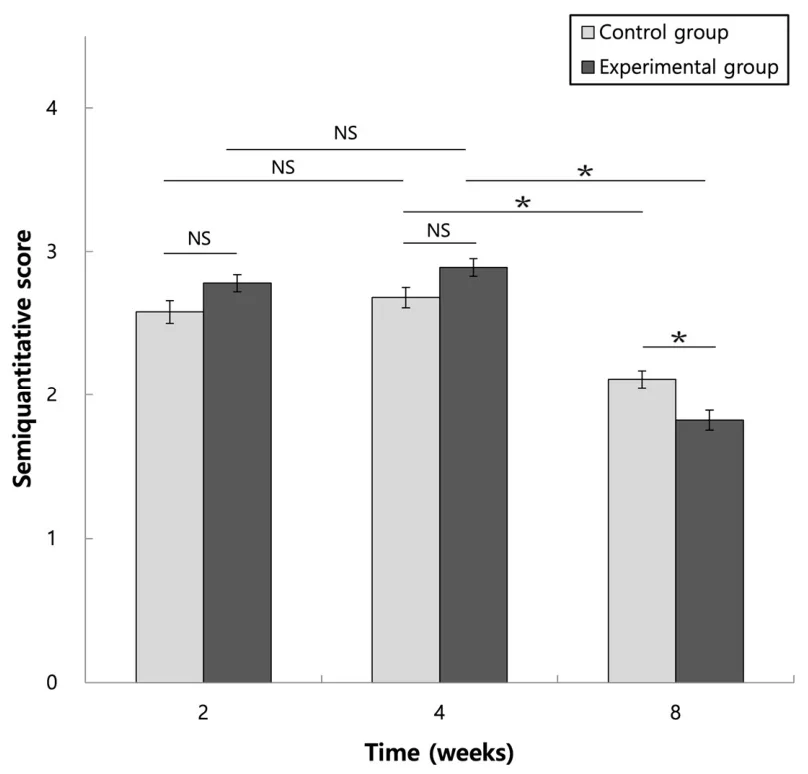
Semiquantitative scoring of BMP7 immunostaining in the control and experimental groups at 2, 4, and 8 weeks. (* *p* < 0.05).

In the control group, the semiquantitative BMP7 expression scores at 2, 4, and 8 weeks were 2.58 ± 0.08, 2.68 ± 0.07, and 2.11 ± 0.06, respectively. For the experimental group, BMP7 scores at the same time points were 2.78 ± 0.06, 2.89 ± 0.06, and 1.82 ± 0.07, respectively. At 2 or 4 weeks, BMP7 expression was significantly higher than at 8 weeks in both groups (*p* < 0.05). At 2 and 4 weeks, BMP7 expression did not differ significantly between the experimental and control groups. At 8 weeks, the experimental group showed significantly lower BMP7 expression than in the control group (*p* < 0.05) (Figure 19).

### 3.10. Expression of Osteocalcin

**Figure 20:**
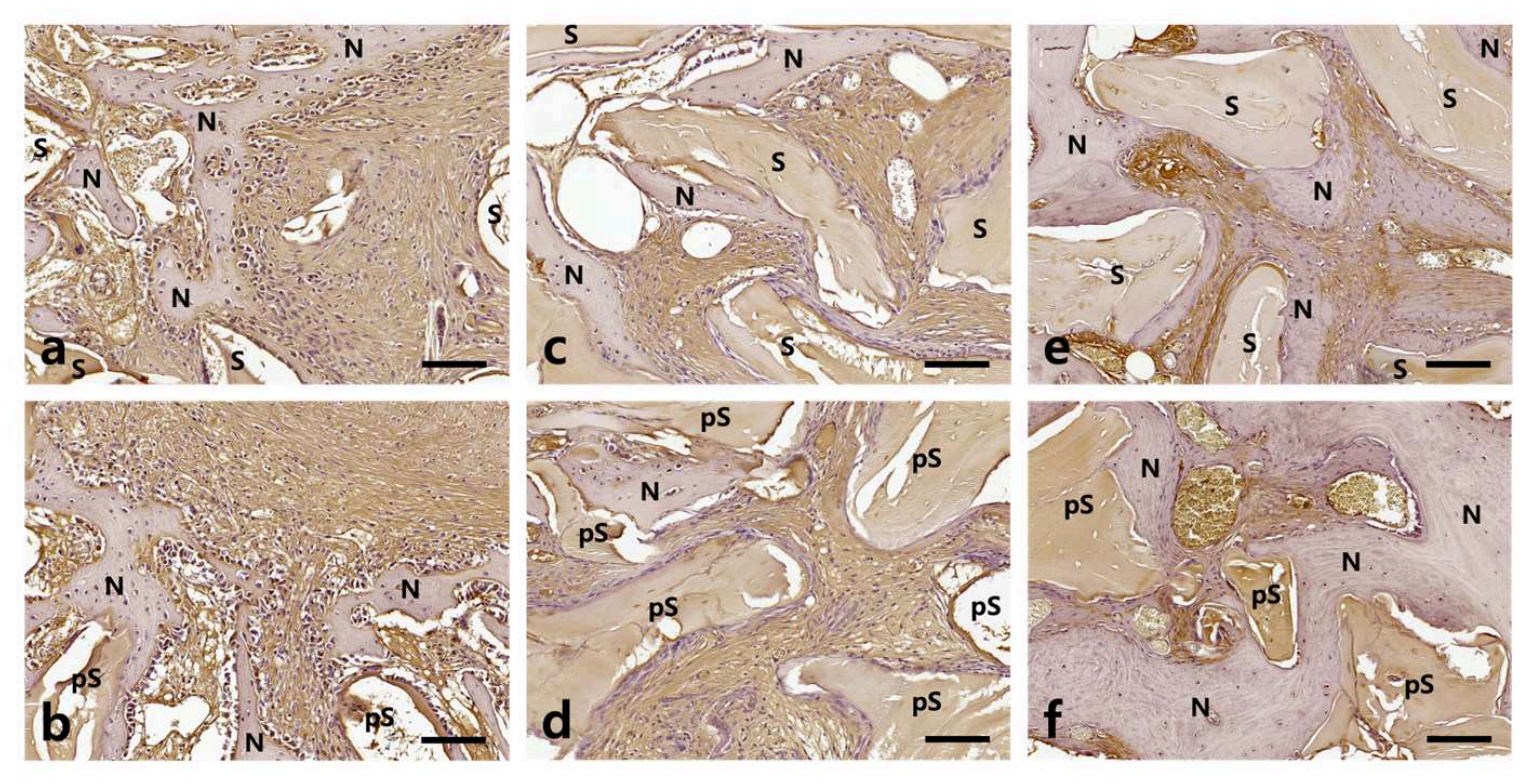
Immunohistochemical staining for osteocalcin in control (**a**,**c**,**e**) and experimental (**b**,**d**,**f**) groups at 2 (**a**,**b**), 4 (**c**,**d**), and 8 (**e**,**f**) weeks after surgery. Abbreviations: N, newly formed bone; S, SANTA-OSS particles; pS, plasma-treated SANTA-OSS particles (Scale bar: 100 μm).

In the control specimens, at 2 weeks after surgery, moderate osteocalcin expression was detected along the surfaces of the newly formed bone and the SANTA-OSS particles and within the surrounding connective tissue. Some positive expression was also detected in osteoblasts along the newly formed bone surface (Figure 20a). At 4 and 8 weeks after surgery, osteocalcin expression decreased, with mild expression detected along the surface of the SANTA-OSS particles and the newly formed bone and within the surrounding connective tissue (Figure 20c,e). In the experimental specimens, at 2 weeks after surgery, strong osteocalcin expression was detected along the surfaces of the newly formed bone and the plasma-treated SANTA-OSS particles, and within fibroblast-rich dense connective tissue. Many osteoblasts located along the newly formed bone surface showed positive expression (Figure 20b). At 4 weeks after surgery, osteocalcin expression decreased, with moderate expression detected along the surface of the newly formed bone and the plasma-treated SANTA-OSS particles and within the surrounding connective tissue (Figure 20d). At 8 weeks after surgery, weak osteocalcin expression was detected along the newly formed bone surface and within the surrounding connective tissue (Figure 20f).

**Figure 21:**
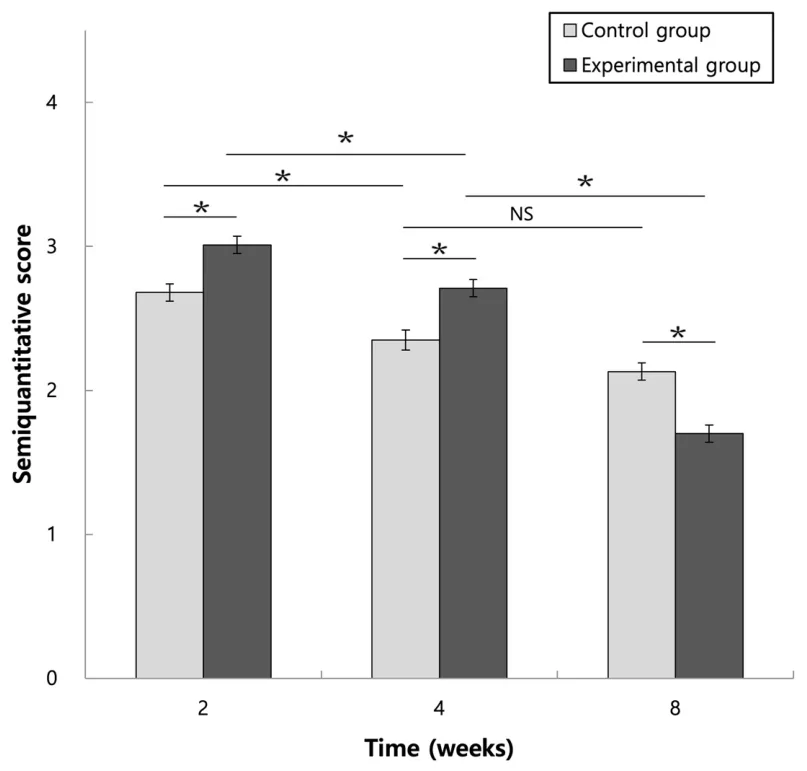
Semiquantitative scoring of osteocalcin immunostaining in the control and experimental groups at 2, 4, and 8 weeks. (* *p* < 0.05).

In the control group, the semiquantitative osteocalcin expression scores at 2, 4, and 8 weeks were 2.68 ± 0.06, 2.35 ± 0.07, and 2.13 ± 0.06, respectively. For the experimental group, osteocalcin scores at the same time points were 3.01 ± 0.06, 2.71 ± 0.06, and 1.70 ± 0.06, respectively. At 2 weeks, osteocalcin expression was significantly higher than at 4 and 8 weeks in both groups (*p* < 0.05). The experimental group showed significantly higher osteocalcin expression than the control group at 2 and 4 weeks. At 8 weeks, the experimental group showed significantly lower osteocalcin expression than in the control group (*p* < 0.05, Figure 21).

### 3.11. Expression of Osteopontin

**Figure 22:**
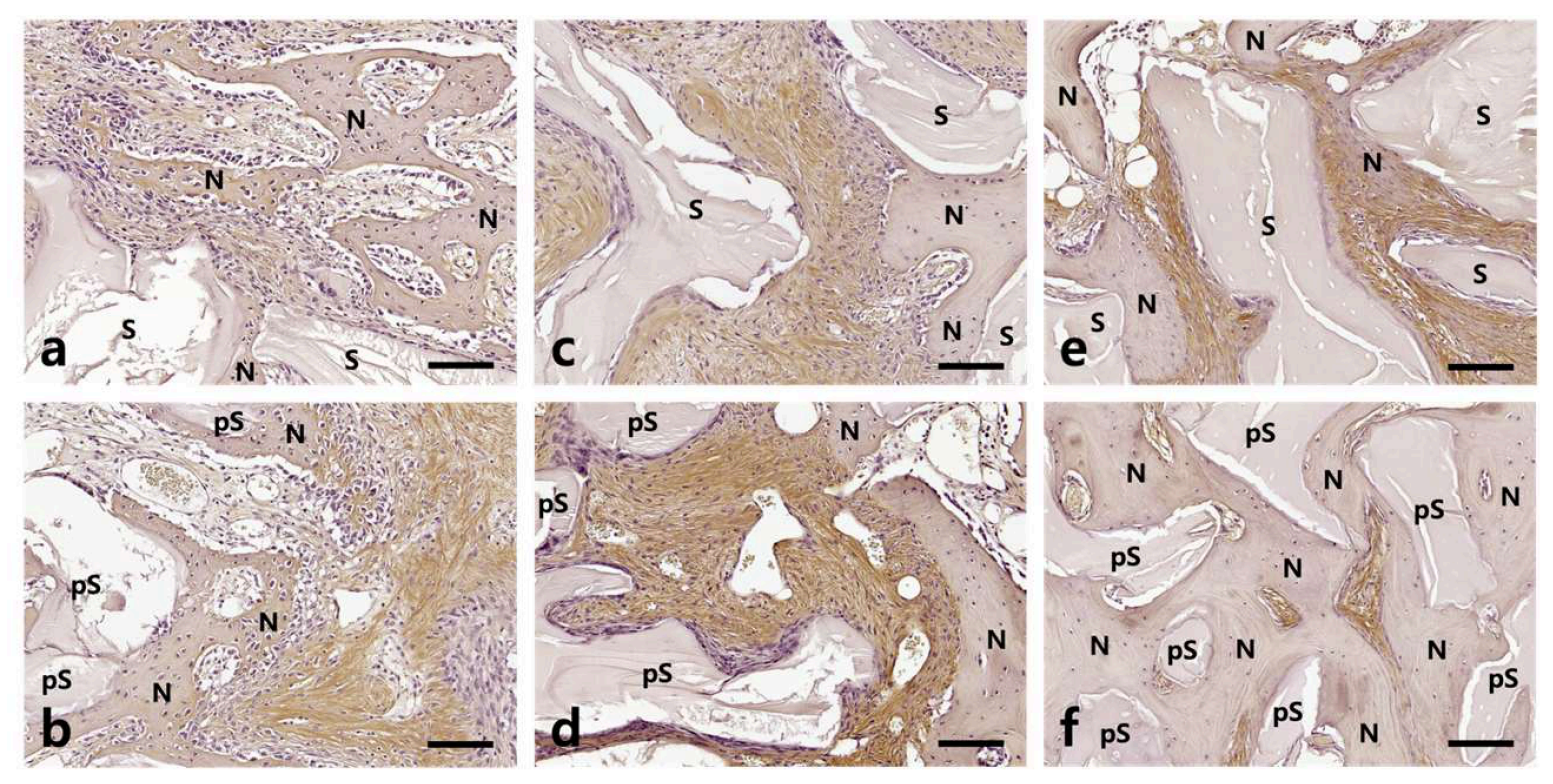
Immunohistochemical staining for osteopontin in control (**a**,**c**,**e**) and experimental (**b**,**d**,**f**) groups at 2 (**a**,**b**), 4 (**c**,**d**), and 8 (**e**,**f**) weeks after surgery. Abbreviations: N, newly formed bone; S, SANTA-OSS particles; pS, plasma-treated SANTA-OSS particles (Scale bar: 100 μm).

In the control specimens, at 2 weeks after surgery, weak osteopontin expression was detected along the surfaces of the newly formed bone and the SANTA-OSS particles and within the surrounding connective tissue (Figure 22a). At 4 and 8 weeks after surgery, osteopontin expression increased, with moderate expression detected along the surface of the newly formed bone and the SANTA-OSS particles and within the surrounding connective tissue (Figure 22c,e). In the experimental specimens, at 2 weeks after surgery, moderate osteopontin expression was detected along the surface of the newly formed bone and the plasma-treated SANTA-OSS particles and within the surrounding connective tissue (Figure 22b). At 4 weeks after surgery, osteopontin expression increased, with strong expression detected within the surrounding connective tissue and along the surface of the newly formed bone and the plasma-treated SANTA-OSS particles (Figure 22d). At 8 weeks after surgery, osteopontin expression decreased, with weak expression detected along the newly formed bone surface and within the surrounding connective tissue (Figure 22f).

**Figure 23:**
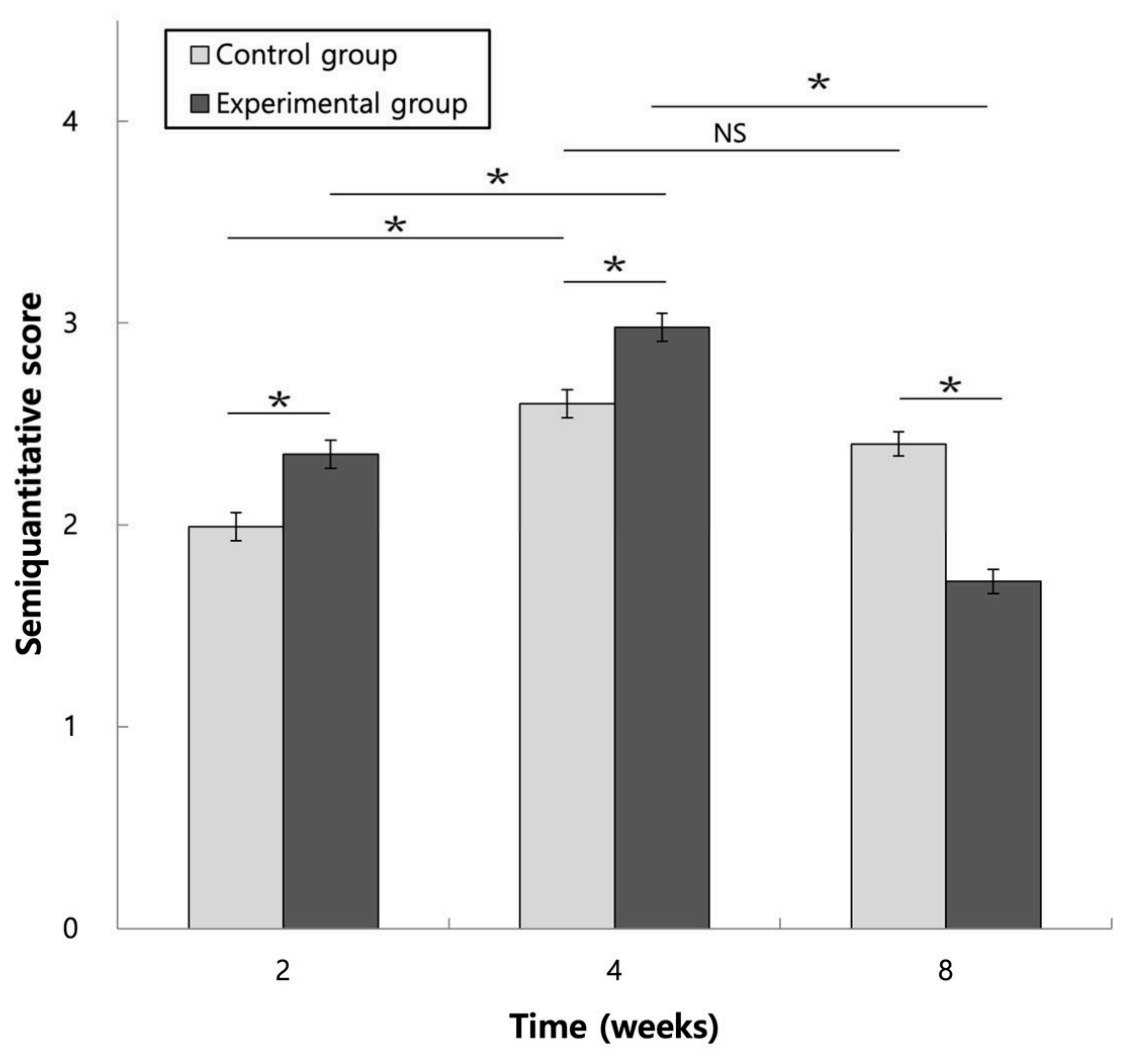
Semiquantitative scoring of osteopontin immunostaining in the control and experimental groups at 2, 4, and 8 weeks. (* *p* < 0.05).

In the control group, the semiquantitative osteopontin expression scores at 2, 4, and 8 weeks were 1.99 ± 0.07, 2.60 ± 0.07, and 2.40 ± 0.06, respectively. For the experimental group, osteopontin scores at the same time points were 2.35 ± 0.07, 2.98 ± 0.07, and 1.72 ± 0.06, respectively. At 4 weeks, osteopontin expression was significantly higher than at 2 and 8 weeks in both groups (*p* < 0.05). The experimental group showed significantly higher osteopontin expression than the control group at 2 and 4 weeks. At 8 weeks, the experimental group showed significantly lower osteopontin expression than the control group (*p* < 0.05) (Figure 23).

## 4. Discussion

Biomaterials, including dental implants and bone graft materials, are typically stored and distributed for a period after manufacturing and sterilization before clinical use, during which airborne hydrocarbons may accumulate on their surfaces. This surface-related biological aging can reduce hydrophilicity and biological activity, potentially compromising protein adsorption and early cellular responses. Ultraviolet (UV) irradiation and plasma treatment have been investigated as approaches to mitigate these surface changes. Among these approaches, non-thermal plasma treatment has been increasingly studied for biomaterial surface modification because it can remove accumulated hydrocarbons and enhance surface hydrophilicity while preserving the underlying physical structure [8,9,10,11,12]. These surface-modifying properties provided the rationale for selecting non-thermal plasma as the surface-treatment approach for the xenograft in this study.

In our previous study, we implanted non-thermal plasma-treated xenografts into rabbit calvarial defects and evaluated them using histological and histomorphometric analyses compared with untreated controls. The plasma-treated group showed enhanced new bone formation from the early stage of healing, with significantly greater new bone and vascular areas than the control group at 2, 4, and 8 weeks [13]. In this study, tissues obtained from the same animal experiment were subjected to immunohistochemical analysis to further evaluate the biological responses associated with these histological and histomorphometric findings. Changes in the expression of multiple markers related to osteogenesis and angiogenesis were particularly evident during the early and intermediate stages of healing. These immunohistochemical findings provide additional biological evidence supporting the increased new bone formation and vascularization observed following non-thermal plasma treatment in our previous study.

Critical-size defects of approximately 10–15 mm have been reported in rabbit calvarial models [14]. In this study, we selected an 8-mm defect diameter to allow two separate defects to be created within the same calvarium while maintaining sufficient spacing between sites.

Among the biological processes involved in bone formation, cell proliferation plays an important role in the early healing stage. PCNA is a nuclear protein that functions as a cofactor for DNA polymerase during DNA replication and is widely used as an immunohistochemical marker of proliferative activity because it is expressed in proliferating cells throughout the cell cycle [15,16,17]. In this study, the plasma-treated experimental group showed significantly greater PCNA expression than the control group at both 2 and 4 weeks, with the highest expression at 4 weeks. In contrast, PCNA expression decreased at 8 weeks, with the experimental group showing significantly lower expression than the control group. This temporal expression pattern indicates that cell proliferation-related responses were more active during the early and intermediate healing stages following plasma application and subsequently decreased at the later stage.

The higher PCNA expression observed during the early and intermediate healing stages may be associated with the enhanced early new bone formation reported in our previous study. The experimental group exhibited significantly greater new bone formation than the control group as early as 2 weeks, and this difference persisted at 4 and 8 weeks [13]. Thus, the higher PCNA expression during the early and intermediate stages supports an association between increased proliferative activity following plasma application and the enhanced new bone formation demonstrated in our previous study. Notably, although the experimental group showed lower PCNA expression than the control group at 8 weeks, new bone formation remained significantly greater in the experimental group in our previous study [13]. Taken together, these findings suggest that cell proliferation-related responses were more active during the early and intermediate healing stages in the experimental group and subsequently decreased at the later stage. A similar temporal pattern was observed by Yamada et al. [18], who reported increased PCNA expression and a higher proportion of PCNA-positive cells during the early period following plasma treatment, followed by a subsequent decrease to levels below those of the control group at the later stage.

Along with cell proliferation, angiogenesis plays an important role in bone regeneration. Newly formed blood vessels supply oxygen and nutrients to bone defect sites and facilitate the recruitment of osteogenic cells, playing an essential role in bone formation. VEGF is a major regulator of angiogenesis, promoting the proliferation and differentiation of vascular endothelial cells [19,20], whereas PECAM-1 is expressed in vascular endothelial cells and is widely used as a histological marker to evaluate angiogenesis [21,22]. In this study, VEGF and PECAM-1 expression was significantly higher in the plasma-treated experimental group than in the control group at 2 and 4 weeks. At 8 weeks, VEGF and PECAM-1 expression did not differ significantly between groups.

These immunohistochemical findings support the increased vascular formation observed in our previous study. In that study, the experimental group exhibited a significantly greater vascular area than the control group at 2, 4, and 8 weeks, with increased vascular formation evident from the early stage of healing [13]. Thus, the higher VEGF and PECAM-1 expression at 2 and 4 weeks is temporally consistent with the increased vascular formation observed from the early healing stage in our previous study [13]. Given the close relationship between angiogenesis and osteogenesis [19,20], this early angiogenic response may also be associated with the increased new bone formation observed in our previous study [13].

After the early phases of cell proliferation and angiogenesis, bone regeneration progresses through osteogenic cell differentiation and the formation and maturation of the bone matrix. In this study, we evaluated the expression of collagen I, collagen III, ALP, BMP2, BMP4, BMP7, osteocalcin, and osteopontin in relation to these osteogenic processes.

Collagen I is a major organic component of the bone matrix and plays an important role in bone formation and structural stability, whereas collagen III contributes to collagen I fibril formation and organization and is associated with bone regeneration and early remodeling processes [23,24,25]. In this study, collagen I and collagen III expression was significantly higher in the plasma-treated experimental group than in the control group at 2 and 4 weeks, whereas the opposite pattern was observed at 8 weeks, with significantly higher expression in the control group. This temporal expression pattern indicates that responses associated with bone matrix formation and remodeling were more active during the early and intermediate healing stages in the experimental group and subsequently decreased at the later stage. The higher expression of collagen I and collagen III during the early and intermediate stages is consistent with the enhanced new bone formation observed in the experimental group from the early healing stage in our previous study [13]. Like the temporal expression pattern observed for PCNA, the lower expression of collagen I and collagen III in the experimental group than in the control group at 8 weeks may reflect temporal changes in matrix-related marker expression during the later stage of healing and remodeling. A similar temporal pattern was observed by Yamada et al. [18], who reported enhanced early collagen I formation following plasma treatment, followed by a subsequent increase in collagen I formation in the control group, and by Miedel et al. [24], who reported decreased collagen III in mature lamellar bone.

ALP is a representative marker expressed during the early stages of osteoblastic differentiation and plays an important role in bone matrix mineralization by hydrolyzing inorganic pyrophosphate and providing inorganic phosphate required for hydroxyapatite formation [26]. BMP family proteins are important signaling molecules that regulate osteoblastic differentiation and bone formation, and BMP signaling promotes osteoblastic differentiation by activating osteogenic transcription factors, including RUNX2. BMP2, BMP4, and BMP7, which were evaluated in this study, are major osteogenic factors closely associated with osteoblastic differentiation and bone formation [27,28]. In this study, ALP, BMP2, and BMP4 expression were significantly higher in the plasma-treated experimental group than in the control group at 2 and 4 weeks. BMP7 expression was also higher in the experimental group at 2 and 4 weeks, although the differences were not statistically significant. At 8 weeks, no significant difference in ALP expression was observed between the groups, whereas BMP2, BMP4, and BMP7 expression was significantly lower in the experimental group than in the control group. These temporal expression patterns indicate that responses associated with osteoblastic differentiation and bone formation were more active during the early and intermediate healing stages in the experimental group and subsequently decreased at the later stage.

The higher expression of ALP, BMP2, and BMP4 during the early and intermediate stages, together with the similar numerical trend in BMP7 expression, is consistent with the enhanced new bone formation observed in our previous study [13]. Similar to the temporal expression patterns observed for PCNA, collagen I, and collagen III, the disappearance of the intergroup difference in ALP expression and the lower expression of BMP2, BMP4, and BMP7 in the experimental group at 8 weeks may reflect temporal changes in osteogenic marker expression during the later stage of healing and remodeling. This temporal interpretation is consistent with Tominami et al. [29], who reported that non-thermal plasma could enhance ALP activity and osteoblastic differentiation, and with Dumic-Cule et al. [30], who described stage-dependent changes in the expression of BMP2, BMP4, and BMP7 during bone healing.

Osteocalcin and osteopontin are representative non-collagenous proteins of the bone matrix and play roles in bone formation as well as in bone matrix mineralization, maturation, and remodeling. Osteocalcin is expressed by osteoblasts and is involved in bone matrix mineralization and maturation, whereas osteopontin mediates interactions between cells and the bone matrix and is associated with bone formation, mineralization, and remodeling [31,32,33]. In this study, expression of both osteocalcin and osteopontin was significantly higher in the plasma-treated experimental group than in the control group at 2 and 4 weeks, whereas the opposite pattern was observed at 8 weeks, with significantly higher expression in the control group. Osteocalcin expression was highest at 2 weeks in both groups and then decreased, whereas osteopontin expression peaked at 4 weeks and decreased at 8 weeks. These temporal expression patterns indicate that responses associated with bone matrix formation, mineralization, and remodeling were more active during the early and intermediate healing stages in the experimental group and subsequently decreased at the later stage.

The higher expression of osteocalcin and osteopontin during the early and intermediate stages is consistent with the enhanced new bone formation observed in our previous study [13]. Similar to the temporal expression patterns observed for PCNA, collagen I, collagen III, and BMP family markers, the lower expression of osteocalcin and osteopontin in the experimental group than in the control group at 8 weeks may reflect temporal changes in marker expression related to bone formation, matrix maturation, and remodeling. This temporal interpretation is consistent with the findings of Ali et al. [34], who reported that osteocalcin and osteopontin expression was associated with normal fracture healing. In addition, Pitol-Palin et al. [35] described osteopontin as a marker of early biomineralization and osteocalcin as a marker associated with later-stage mineralization and bone maturation, further supporting this interpretation.

The immunohistochemical markers evaluated in this study generally showed a temporal pattern characterized by higher expression in the experimental group during the early and intermediate healing stages, followed by decreased expression, disappearance of intergroup differences, or reversal of these differences at the later stage. In our previous study, the experimental group showed increased new bone formation and vascularization from the early healing stage, while at 8 weeks, graft resorption and bone marrow formation were greater than in the control group [13]. In addition, the experimental group showed a significantly higher number of TRAP-positive osteoclasts than the control group at 2, 4, and 8 weeks, with the highest level at 4 weeks, supporting increased activity associated with graft resorption and bone remodeling [13]. Taken together, these findings suggest that biological responses associated with bone regeneration were more active during the early and intermediate healing stages in the experimental group and subsequently changed during the later stage of healing and remodeling. However, the lower expression of several markers at 8 weeks should not be interpreted as direct evidence of accelerated tissue maturation.

These immunohistochemical findings may be interpreted in light of previous studies on the effects of non-thermal plasma on biomaterial surface properties. Non-thermal plasma treatment can reduce carbon-based contaminants on material surfaces and improve hydrophilicity and wettability, thereby creating surface conditions favorable for cell adhesion and osteogenic differentiation [12,17]. The changes observed in several markers associated with cell proliferation, angiogenesis, and osteogenesis during the early healing stage in this study suggest a possible biological link between plasma-induced changes in surface properties and the enhanced bone regeneration observed in our previous study.

This study has several limitations. First, we assessed immunohistochemical expression using semiquantitative scoring, which limits absolute quantification of individual protein expression levels. Second, we evaluated only three time points (2, 4, and 8 weeks); therefore, we could not fully characterize the temporal expression profile of each marker. Third, the rabbit calvarial model is biologically relevant to alveolar bone regeneration because both sites undergo intramembranous bone formation [36]. Nevertheless, bone healing proceeds more rapidly in rabbits than in humans [37], and the calvarial model does not fully reproduce the clinical environment of the human alveolar ridge. Therefore, caution is warranted when translating these findings to human alveolar bone regeneration. Fourth, chemical changes in the graft surface after plasma treatment and intracellular signaling pathways were not directly analyzed; therefore, a direct causal relationship between specific surface modifications and changes in the expression of individual immunohistochemical markers could not be established. Fifth, although the control and experimental defects were created contralaterally within the same animal, the within-animal pairing was not separately accounted for in the statistical analysis. This statistical limitation should be considered when interpreting the between-group comparisons. Further studies incorporating quantitative protein assays and gene expression analyses are needed to better characterize the osteogenic and angiogenic responses and their underlying mechanisms following plasma treatment. Sixth, only one plasma-treatment protocol (two consecutive 1-min cycles) was evaluated; therefore, the influence of different treatment durations or numbers of cycles on the biological responses could not be determined. The present findings should therefore be interpreted within the context of the treatment parameters used in this study.

## 5. Conclusions

Non-thermal plasma treatment of xenogenic bone grafts was associated with temporal changes in immunohistochemical markers related to cell proliferation, angiogenesis, and osteogenesis, with differences between the experimental and control groups occurring predominantly during the early and intermediate healing stages. These findings indicate distinct biological responses around plasma-treated xenografts during bone regeneration.

## Data Availability

The original contributions presented in this study are included in the article. Further inquiries can be directed to the corresponding author.
